# Omomyc Reveals New Mechanisms To Inhibit the MYC Oncogene

**DOI:** 10.1128/MCB.00248-19

**Published:** 2019-10-28

**Authors:** Mark J. Demma, Claudio Mapelli, Angie Sun, Smaranda Bodea, Benjamin Ruprecht, Sarah Javaid, Derek Wiswell, Eric Muise, Shiying Chen, John Zelina, Federica Orvieto, Alessia Santoprete, Simona Altezza, Federica Tucci, Enrique Escandon, Brian Hall, Kallol Ray, Abbas Walji, Jennifer O’Neil

**Affiliations:** a Oncology Discovery, Merck & Co., Inc., Boston, Massachusetts, USA; b Discovery Chemistry, Merck & Co., Inc., Kenilworth, New Jersey, USA; c Protein Science, Merck & Co., Inc., Boston, Massachusetts, USA; d Chemical Biology, Merck & Co., Inc., Boston, Massachusetts, USA; e Genetics and Pharmacogenomics, Merck & Co., Inc., Boston, Massachusetts, USA; f Assay Development, Merck & Co., Inc., Palo Alto, California, USA; g Pharmokinetics, Merck & Co., Inc., Kenilworth, New Jersey, USA; h IRBM, SpA, Pomezia, RM, Italy

**Keywords:** DNA binding, Max, Myc, Omomyc, cotranslational, rRNA, transcription

## Abstract

The MYC oncogene is upregulated in human cancers by translocation, amplification, and mutation of cellular pathways that regulate Myc. Myc/Max heterodimers bind to E box sequences in the promoter regions of genes and activate transcription. The MYC inhibitor Omomyc can reduce the ability of MYC to bind specific box sequences in promoters of MYC target genes by binding directly to E box sequences as demonstrated by chromatin immunoprecipitation (CHIP). Here, we demonstrate by both a proximity ligation assay (PLA) and double chromatin immunoprecipitation (ReCHIP) that Omomyc preferentially binds to Max, not Myc, to mediate inhibition of MYC-mediated transcription by replacing MYC/MAX heterodimers with Omomyc/MAX heterodimers. The formation of Myc/Max and Omomyc/Max heterodimers occurs cotranslationally; Myc, Max, and Omomyc can interact with ribosomes and Max RNA under conditions in which ribosomes are intact. Taken together, our data suggest that the mechanism of action of Omomyc is to bind DNA as either a homodimer or a heterodimer with Max that is formed cotranslationally, revealing a novel mechanism to inhibit the MYC oncogene. We find that *in vivo*, Omomyc distributes quickly to kidneys and liver and has a short effective half-life in plasma, which could limit its use *in vivo*.

## Abstract (precis)

The MYC oncogene is upregulated in human cancers by translocation, amplification, and mutation of cellular pathways that regulate Myc. Myc/Max heterodimers bind to E box sequences in the promoter regions of genes and activate transcription.

## INTRODUCTION

The MYC protein is encoded by an oncogene that is activated by amplification, chromosomal translocation, activation of upstream signaling pathways, and mutation of pathways involved in regulating MYC protein stability (1–3). MYC is a basic helix-loop-helix (bHLH) protein that acts as a transcriptional activator. The amino terminus of MYC contains a transcriptional activation domain and can interact with various factors involved in transcriptional activation and epigenetic factors (1–4). The carboxy terminus of MYC contains a helix-loop-helix domain, a leucine zipper domain, and a DNA binding domain. Although Myc by itself can bind to a DNA sequence, CACGTG, referred to as an E box (1–3, 5, 6), for high-affinity binding to E box DNA, Myc forms a heterodimer via its bHLH and leucine zipper domains with its partner protein Max, which is required for MYC-induced cellular transformation (7, 8). The E box sequence is present in approximately 15% of all cellular loci (9), indicating that the Myc/Max heterodimer can potentially control the transcriptional activation of many genes, which vary in their affinities for binding MYC (9). What defines a “high-affinity” site may be the presence of other accessory proteins, such as WD repeat-containing protein 5 (WDR5), which can bind to MYC and help stabilize its interaction with DNA (10, 11). When the interaction between WDR5 and MYC is disrupted, there is an 80% attenuation of MYC binding to its chromosomal locations and disruption of MYC-driven tumorigenesis (10). In addition, overexpression of MYC changes the transcriptional program of MYC, likely due to binding “low-affinity” sites that are not bound when MYC is expressed at normal levels (11).

Various strategies to inhibit MYC have been attempted, including inhibition of either Myc expression (12) or Myc/Max dimerization (12). A cellular antagonist of Myc, the protein Mad1 (Mxd1), also binds to Max and antagonizes Myc transcriptional activity. Mad1/Max dimers directly compete with Myc/Max dimers for access to E boxes in target promoters (13–17). Mad1 also recruits the mSin3 repressor complex to inhibit promoter activation (13, 18). Mad1 competes with Myc to bind upstream binding factor (UBF), thereby repressing ribosomal subunit expression in the nucleolus, with the result of inhibiting both cell proliferation and Myc-mediated transformation (13, 17, 19).

The most studied Myc/Max inhibitor is Omomyc, a miniprotein derived from the bHLH domain of Myc, with several mutations in the leucine zipper to improve dimerization (20). When expressed in cells, Omomyc can inhibit MYC-mediated transcription and inhibit cell proliferation (20). In addition, Omomyc can be immunoprecipitated with Myc and Max, as well as Miz1, a zinc finger protein that can interact with Myc on promoters but does not interact with other HLH proteins, such as hypoxia-inducible factor 1α (HIF-1α) (21). In a Myc-inducible mouse tumor model, the expression of Omomyc resulted in an increased survival rate compared to that of control mice (22). Side effects in highly proliferative tissues were mild and fully reversible, indicating that Omomyc could be a relatively safe treatment for tumors with MYC overexpression (22).

Reports have suggested that recombinant Omomyc can bind to E box DNA with an affinity similar to those of recombinant MYC and MAX homo- and heterodimers as well as to a MYC/Omomyc heterodimer *in vitro* (23). Chromatin immunoprecipitation (CHIP) experiments with cells in which Omomyc is ectopically overexpressed show that Omomyc can reduce the amount of MYC binding to promoters, and Omomyc can bind promoters itself, suggesting that Omomyc binds to DNA and prevents the MYC/MAX heterodimer from binding to DNA (23). Similarly, a hybrid protein, ME-47 (Max DNA binding domain, dimerization domain of bHLH protein E47), has also been shown to bind E boxes when ectopically expressed and to block the ability of Myc/Max heterodimers to bind DNA (12, 24, 25). Beaulieu et al. recently showed that recombinant Omomyc is cell penetrant, can disrupt MYC transcriptional regulation by reducing the amount of Myc protein that could interact with promoters, and has *in vivo* activity (26). In addition, they showed that recombinant Omomyc can form stable homodimers or heterodimers with recombinant Max *in vitro*.

Here, we show that treatment of cells with recombinant Omomyc leads to an overall reduction in the levels of Myc protein. This reduction of Myc protein is due to the ubiquitination and degradation of Myc, leading to a reduction in Myc/Max heterodimer formation as judged by immunoprecipitation and proximity ligation assays (PLAs). PLA results also show that Omomyc can form dimers with both Max and differentially labeled Omomyc monomers in the cell. We also show that Omomyc can also bind to DNA as either a homodimer or a heterodimer with Max but not as a heterodimer with Myc, as determined by both CHIP and double chromatin immunoprecipitation (ReCHIP) experiments. Both translating ribosome affinity purification (TRAP) and RNA immunoprecipitation (RIP) experiments indicate that Myc, Omomyc, and Max can interact with ribosomes and Max RNA under conditions where ribosomes are intact, indicating that dimerization of these proteins with Max occurs cotranslationally. Although active with cells in culture, we found that Omomyc had suboptimal pharmacokinetic (PK) properties that lead to a short half-life in mice.

Our data suggest that besides binding DNA as a homodimer, Omomyc can function similarly to Mad1, where Omomyc competes with Myc for binding to and forming a heterodimer with Max. Omomyc/Max dimers can then bind promoter regions of Myc target genes to repress transcription. Like Mad1/Max dimers, Omomyc/Max dimers also bind to the promoter region of ribosomal subunits in the nucleolus, potentially limiting cell growth by affecting the available pool of ribosomal subunits. Our study suggests that Omomyc has a dual mechanism of action: (i) Omomyc dimers directly compete with Myc/Max heterodimers for E box binding, and (ii) Omomyc competes with Myc for binding to Max cotranslationally, leading to a rebalancing of the cellular Myc/Max ratio and causing a reduction in the levels of Myc protein and repression of Myc-mediated genes by a mode similar to that of Mad1 repression of Myc.

## RESULTS

### Recombinant Omomyc inhibits cancer cell proliferation and affects MYC-mediated transcription.

**FIG 1:**
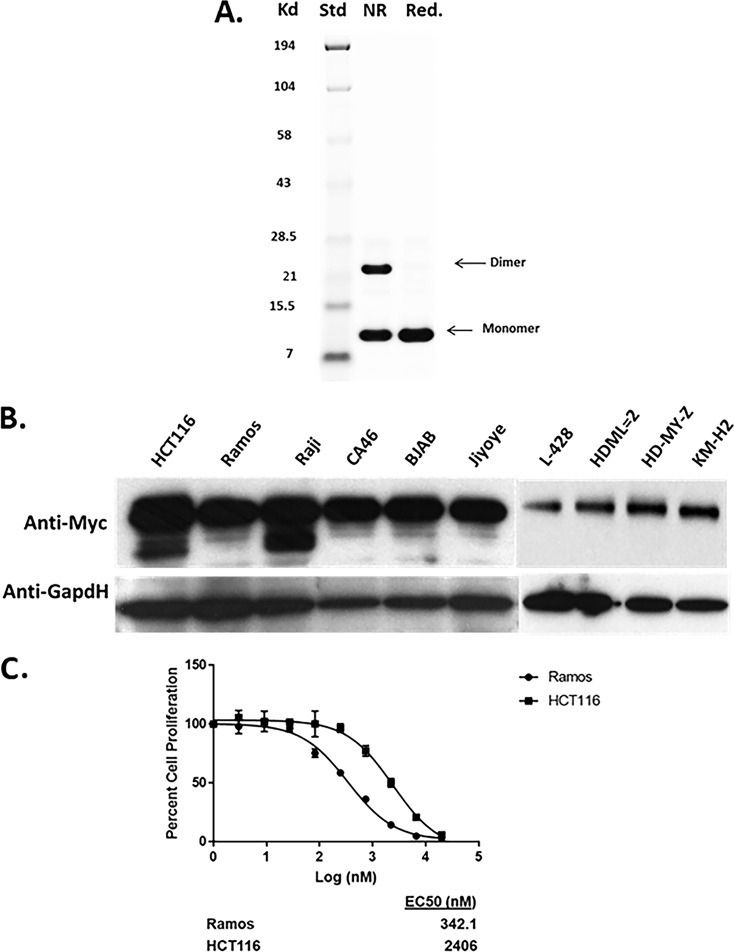

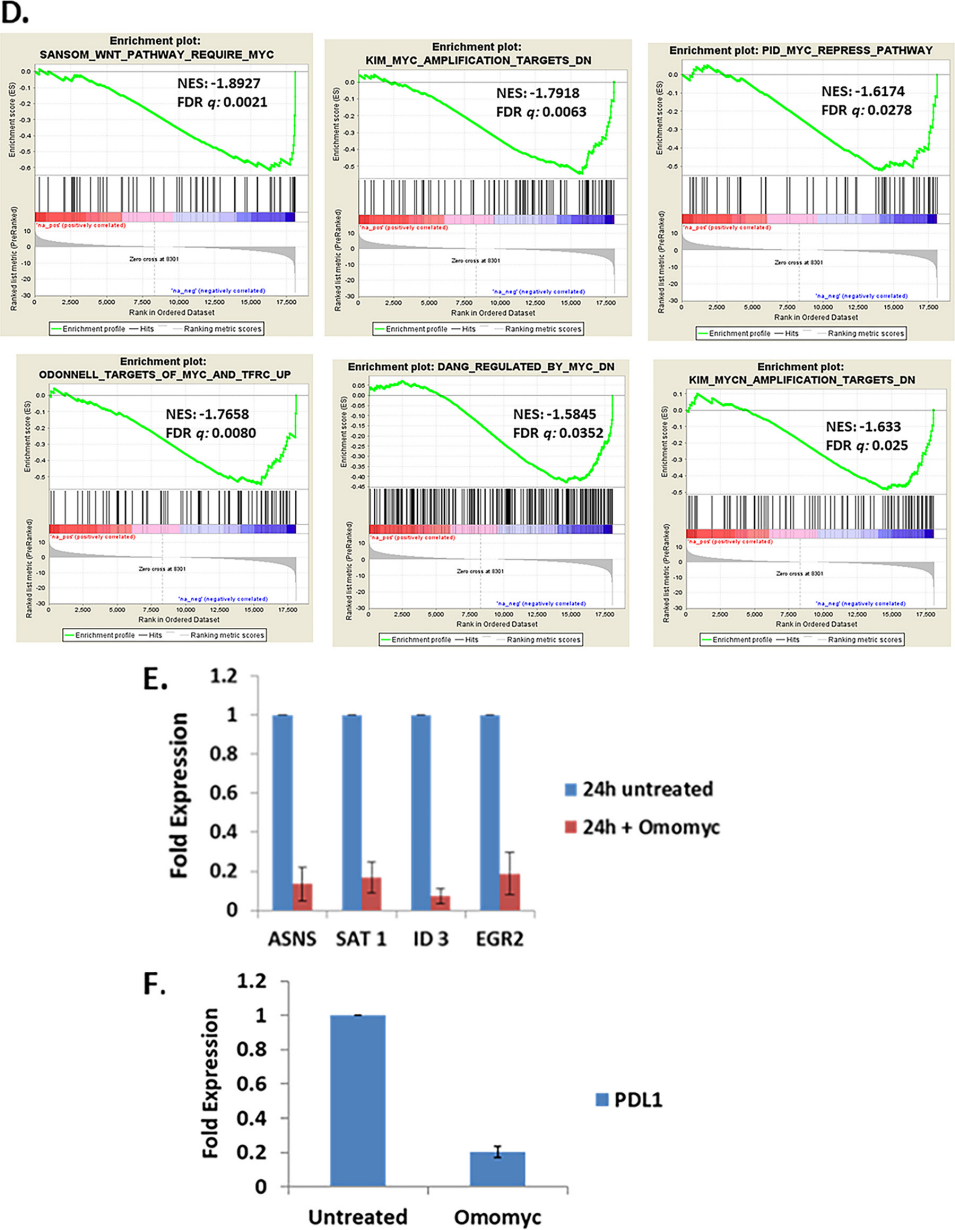
Omomyc affects cell proliferation and MYC-mediated transcription. (A) Purification and characterization of recombinant Omomyc. Shown is an SDS-PAGE gel of bacterially expressed Omomyc under nonreduced (NR) and reduced (Red) conditions. (B) Myc levels of cells used for cell proliferation and other experiments. (C) Effect of both recombinant Omomyc and synthetic Omomyc on proliferation of Ramos and HCT116 cells over 3 days. (D) Gene set enrichment analysis (GSEA) comparing gene expression between untreated and 10 μM Omomyc-treated HCT116 cells. Normalized enrichment scores (NES), false discovery rate (FDR) *q* values, and numbers of genes for MYC signatures are shown. (E and F) Q-PCR showing the effect of 10 μM Omomyc on the expression of several Myc target genes identified by RNA-Seq in HCT116 cells. Genes tested were the ASNS, SAT1, ID3, EGR2, and CD274 (PD-L1) genes.

In order to fully understand the mechanism of action of the Myc inhibitor Omomyc, we expressed the miniprotein in Escherichia coli (Fig. 1A) or synthesized Omomyc using peptide synthesis techniques. Size exclusion chromatography (data not shown) and native gel electrophoresis indicated that Omomyc was present as a dimer and a monomer in solution (Fig. 1A). Once purified, recombinant or synthetic Omomyc was used to treat cell lines in which Myc is either amplified or stabilized and which have high protein levels (Fig. 1B). Both Ramos lymphoma cells with a Myc translocation and HCT116 colon cancer cells in which Myc is stabilized show sensitivity to Omomyc in a 72-h cell proliferation assay (50% inhibitory concentration [IC_50_] of ∼400 nM for Ramos cells and IC_50_ of 2 to 3 μM for HCT116 cells) (Fig. 1C). Similarly, lymphoma cell lines that have a MYC translocation and a high level of Myc protein (Fig. 1B) are sensitive to Omomyc, with a 50% effective concentration (EC_50_) range of 0.4 to 1.1 μM, whereas lymphoma cell lines with low MYC RNA and low Myc protein levels (Fig. 1B) are insensitive to Omomyc (Table 1).

**TABLE 1:** Effect of Omomyc on cell proliferation for Myc-“high” and Myc-“low” lymphoma cell lines^*a*^

Cell line	MYC status	EC_50_ of Omomyc (nM)
Ramos	MYC translocation	∼400–500
Daudi	MYC translocation	1,100
CA46	MYC translocation	500
BJAB	MYC translocation	473
Jiyoye	MYC translocation	1,500
Raji	MYC translocation	1,400
KM-H2	Low MYC RNA	>20,000
L-428	Low MYC RNA	6,300
HDLM-2	Low MYC RNA	>20,000
HD-MY-Z	Low MYC RNA	>20,000

a Cells were plated and assayed as described in Materials and Methods.

As confirmation that Omomyc had cellular activity, we determined if Omomyc could alter the expression profile of HCT116 cells by performing transcriptome sequencing (RNA-Seq) on cells treated with Omomyc. We treated HCT116 cells with 10 μM Omomyc in the presence of ProteoJuice, a protein transfection reagent that forms noncovalent interactions with protein and has endosome-protective properties, ensuring the delivery of intact protein within the cell. Treatment with Omomyc resulted in changes in the transcriptional profile of HCT116 cells at 24 h (Fig. 1D; see also Table SI in the supplemental material), with downregulation of several MYC-driven gene signatures (Fig. 1D and Table 2), along with changes in the overall transcription profile between control cells and treated cells (Table SI). Several of the genes downregulated by Omomyc treatment were identified as downstream effectors of Myc transformation, such as the ASNS and SAT1 metabolism genes, the transcription factor ID3 gene, and the EGR2 immediate early gene (Fig. 1E). We treated HCT116 cells for 24 h with 10 μM Omomyc in the presence of ProteoJuice, and using reverse transcription-PCR (RT-PCR), we confirmed that each of these genes from the RNA-Seq experiment as well as another known Myc target gene, the CD274 gene (27), were significantly downregulated by 24 h (Fig. 1E and F). These results validate that Omomyc has cellular activity and is an inhibitor of Myc-mediated transcriptional activation.

**TABLE 2:** Effect of Omomyc on MYC gene signature gene sets^*a*^

Gene set	No. of genes	NES	FDR *q* value
SANSOM_WNT_PATHWAY_REQUIRE_MYC	55	−1.8927	0.0021
KIM_MYC_AMPLIFICATION_TARGETS_DN	87	−1.7918	0.0063
ODONNELL_TARGETS_OF_MYC_AND_TFRC_UP	78	−1.7658	0.0080
DANG_REGULATED_BY_MYC_DN	240	−1.5845	0.0352
KIM_MYCN_AMPLIFICATION_TARGETS_DN	94	−1.633	0.0250
PID_MYC_REPRESS_PATHWAY	59	−1.6174	0.0278

a Selected MYC gene signatures are from GSEA. NES, normalized enrichment score; FDR, false discovery rate.

### Omomyc binds to MYC and MAX and leads to the destabilization of Myc.

**FIG 2:**
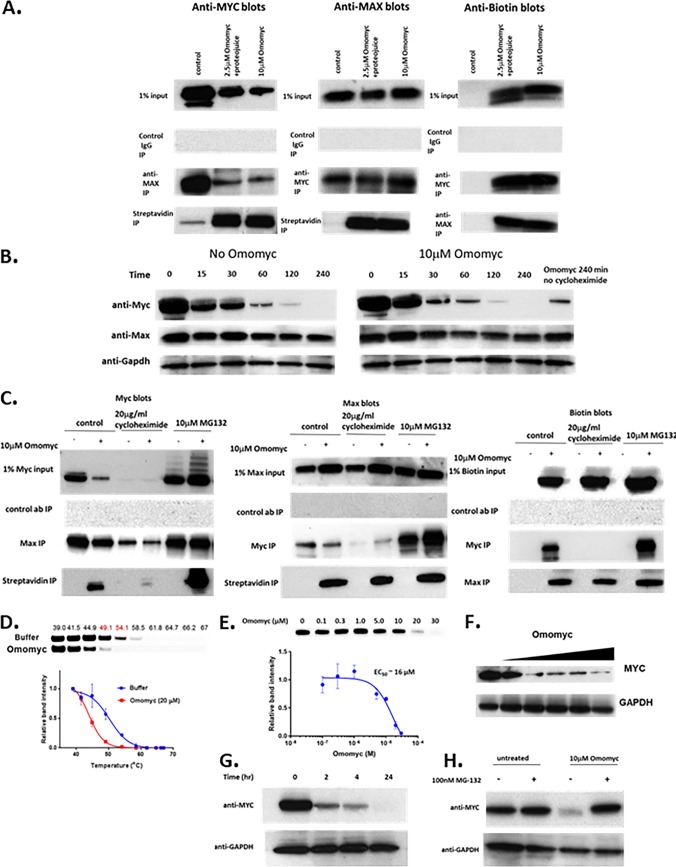
Omomyc interacts with Myc and Max in cells and also affects Myc stability and protein levels. (A) Immunoprecipitation (IP) of Myc, Max, and biotinylated Omomyc in HCT116 cells. HCT116 cells were treated with 10 μM Omomyc, 2.5 μM Omomyc with ProteoJuice, or no Omomyc at all and then lysed after 24 h. Lysates were then incubated with antibodies against Myc and Max, control IgG, and streptavidin; pulled down with magnetic beads; run on an SDS-PAGE gel; and then blotted with antibodies to Myc, Max, or biotin. (B) Effect of treatment with 10 μg/ml cycloheximide in the presence and absence of 10 μM Omomyc over time. HCT116 cells were treated with 10 μg/ml cycloheximide in the presence or absence of 10 μM Omomyc for up to 4 h. Cells were lysed, subjected to SDS-PAGE, and then Western blotted with antibodies to Myc, Max, and Gapdh. (C) Effect of cycloheximide or the proteasome inhibitor MG132 on Myc, Max, and Omomyc interactions. HCT116 cells were treated with 0 or 10 μM Omomyc in the absence or presence of 20 μg/ml cycloheximide or 10 μM MG132 for 4 h; lysed; incubated with antibodies (ab) to Myc and Max, control IgG, and streptavidin; and then pulled down with magnetic beads. The material was run on an SDS-PAGE gel and Western blotted with antibodies to Myc, Max, and biotin. (D) Treatment of Ramos cell lysates with Omomyc induces thermal destabilization of Myc. Shown are aggregation temperature (*T*_agg_) curves and corresponding Western blot images for Myc in Ramos cell lysates after incubation at 4°C with Omomyc (20 μM) or PBS for 2 h. All data were normalized to the Myc band intensity for the lowest temperature, 39°C. The *T*_agg_ shifts were analyzed using the Boltzmann sigmoidal equation and are 50.3°C for the buffer-treated lysate and 43.9°C for the Omomyc-treated lysate. (E) Omomyc decreases the level of Myc in the cell. Ramos cells were treated with increasing concentrations of Omomyc for 24 h, lysed, run on an SDS-PAGE gel, and Western blotted with antibodies to Myc and Gapdh. (F) Time course of Myc reduction by 10 μM Omomyc. Ramos cells were treated with Omomyc, and samples were collected at 0, 2, 4, and 24 h. The samples were lysed, subjected to SDS-PAGE, and Western blotted for Myc and Gapdh. (G) Myc reduction is due to proteosomal degradation of Myc. Ramos cells were treated with or without 10 μM Omomyc in the presence or absence of 100 nM MG132 for 24 h, lysed, subjected to SDS-PAGE, and Western blotted for Myc and Gapdh.

To assess how Omomyc affects Myc/Max binding, we used a biotinylated Omomyc to immunoprecipitate Myc and Max from cells. We treated HCT116 cells with 2.5 μM Omomyc or with 10 μM Omomyc in the presence of ProteoJuice for 24 h. We then lysed the cells and performed immunoprecipitation with anti-Myc, anti-Max, or streptavidin (Fig. 2A). Western blotting of the inputs for the immunoprecipitations showed that upon treatment with Omomyc, there was a reduction in the amount of Myc that could be immunoprecipitated with Max. Both Myc and Max bound to biotinylated Omomyc. In addition, the levels of Max in the input were relatively constant, indicating that Max levels are not affected by Omomyc treatment.

To determine the effect of inhibiting protein synthesis on Myc and Max levels in the presence or absence of Omomyc, we treated HCT116 cells with 10 μg/ml cycloheximide and performed a time course to determine if there was a change in Myc and Max protein levels (Fig. 2B). Myc levels were significantly reduced within 30 min with or without Omomyc, but Max levels remained stable up to 4 h (240 min) (Fig. 2B).

HCT116 cells were treated with cycloheximide in the presence or absence of Omomyc (Fig. 2C). After 4 h, Myc levels were significantly reduced and not stabilized by Omomyc, and there was a significant reduction in the amount of Max or Omomyc that could be immunoprecipitated with Myc. In the input samples with Omomyc treatment, there was an increase in the amount of Max that was not dependent upon cycloheximide treatment (Fig. 2C). Max was immunoprecipitated in a complex with biotinylated Omomyc with or without cycloheximide, while cycloheximide treatment decreased the amount of Myc immunoprecipitated with either Max or biotinylated Omomyc.

Next, to determine if the reduction of Myc levels was due to ubiquitination and proteasomal degradation, we treated cells with the proteasomal inhibitor MG132. Max levels remained unchanged with MG132 treatment and did not show evidence of ubiquitination. With MG132 treatment, the levels of MYC increased with Omomyc treatment, with laddering indicative of the preservation of ubiquitination when proteasomal degradation is inhibited (28). In the presence of MG132, Myc, Max, and biotinylated Omomyc all immunoprecipitated with one another. The levels of interaction between Omomyc and Max are similar in the absence and presence of MG132 (Fig. 2C). However, there appears to be an increase in the amount of Myc that is immunoprecipitated with Omomyc in the presence of MG132. This suggests that Omomyc can interfere with the binding of Myc to Max, leading to the ubiquitination and degradation of the free Myc monomer in the cell. When proteasomal degradation is inhibited, there is Myc stabilization that allows excess Myc to interact with Max.

As confirmation of Omomyc leading to Myc destabilization, we performed a cellular thermal shift assay (CETSA), which is based upon the biophysical principle of ligand-induced stabilization of a target protein (29). In this assay, we used Ramos cell lysates to determine the impact of Omomyc on Myc thermal stabilization. Using 20 μM Omomyc, we observed that there was a significant thermal destabilization of Myc in the Ramos cell lysates, with a change in the aggregation temperature (*T*_agg_) of 6°C (Fig. 2D). By performing an isothermal dose-response fingerprint (ITDRF) experiment at 54°C, we determined the dose dependence of this destabilization, establishing an EC_50_ for Omomyc of ∼16 ± 2 μM (Fig. 2E), confirming that Omomyc can destabilize Myc.

Treatment of Ramos cells with Omomyc for 24 h decreased the level of Myc present in the cell in a dose-dependent manner as determined by Western blotting (Fig. 2F). This reduction in Myc levels was consistent in other cell lines (data not shown) and occurred within 2 h (Fig. 2G). The reduction in Myc levels was inhibited by a proteasome inhibitor, MG132, indicating that Omomyc destabilizes Myc, leading to its degradation by the proteasome (Fig. 2B and H).

### Omomyc binds to E box sequences in DNA and displaces Myc/Max heterodimers.

**FIG 3:**
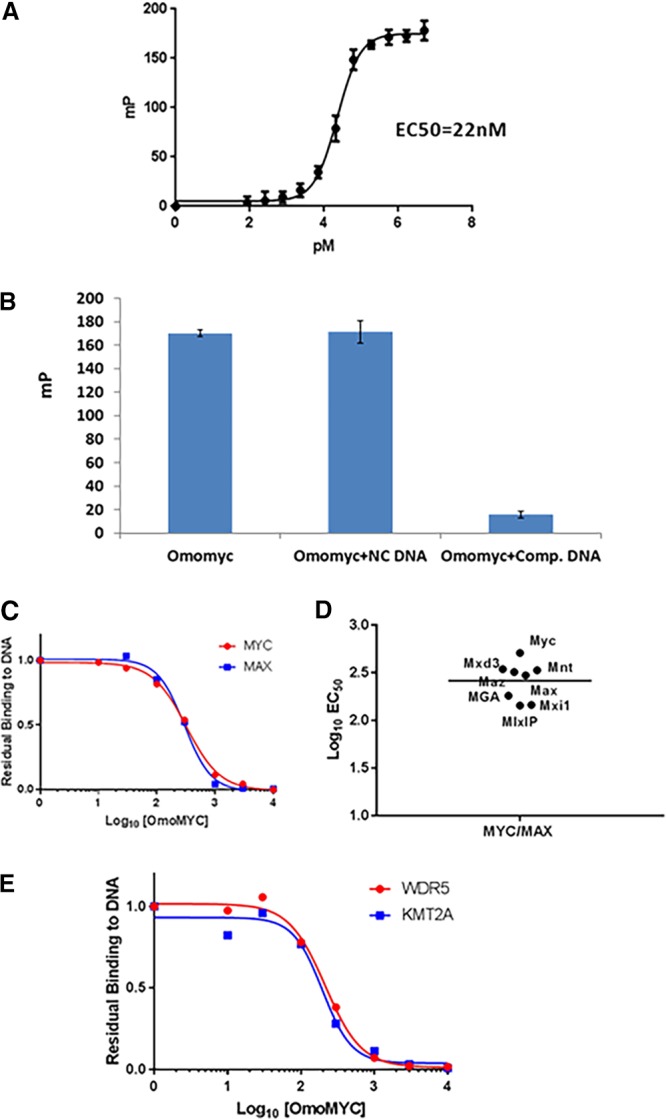
Omomyc binds DNA *in vitro*. (A) Fluorescence polarization assay using 5′-FAM-labeled 20-mer E box DNA and synthetic Omomyc, demonstrating DNA binding activity in millipolarizations (mP) of Omomyc (22 nM). (B) Competitive binding fluorescence polarization assay combining 5′-FAM-labeled Myc E box DNA with either competitive (unlabeled E box) DNA or noncompetitive (scrambled E box) DNA. (C) Dose-resolved proteomic pulldown experiments using immobilized Myc E box DNA. As expected, both MYC and MAX are competed off the DNA with increasing concentrations of free Omomyc spiked into the Ramos cell lysate (Myc EC_50_ = 321 nM; MAX EC_50_ = 297 nM). Owing to the sequence overlap between Omomyc and Myc, we show quantitative values for a representative MYC peptide. The 10 nM Max data point was excluded because it was an obvious outlier. (D) Omomyc can compete for binding to the E box with Myc, Max, and other dimerization partners (Mxi1, Mxd3, Mnt, Mga, and Maz). The experiment was performed using a method similar to the one described above for panel C. All dimerization partners have similar EC_50_s. (E) WDR5 and KMT2A, two core components of the MYC-associated MLL complex, are potently competed by Omomyc (WDR5 EC_50_ = 213 nM; KMT2A EC_50_ = 196 nM).

Previous reports (24) have shown that Omomyc can bind to DNA. We developed an *in vitro* fluorescence polarization (FP) assay to measure the binding of Omomyc to DNA (Fig. 3A). In this assay, Omomyc bound DNA containing the canonical E box sequence, with a *K_d_* (dissociation constant) of approximately 22 nM. Omomyc binding to DNA was specific, since binding could not be competed away with a noncompetitive oligonucleotide but could with a competitive oligonucleotide that contained an intact E box (Fig. 3B). Using E box DNA coupled to beads, we also performed an E box DNA binding pulldown with the Ramos cell lysate with increasing concentrations of Omomyc as a competitor for proteins in the lysate that could bind E box DNA. The proteins pulled down by the E box DNA beads were then subjected to mass spectrometry (MS) analysis (Fig. 3C). We found that Omomyc could effectively compete with Myc and Max for DNA binding in the cell lysate, suggesting that Omomyc can potentially compete with Myc for E box binding in the cell. In addition, Omomyc could also prevent other Myc and Max dimerization partners, such as Mxi1, MGA, and Mxd3, from binding DNA (Fig. 3D). Myc can also associate with proteins in the MLL1 histone methyltransferase complex, namely, WDR5 (10, 11), which helps recruit Myc to chromatin, and KMT2A, a histone methyltransferase in the MLL1 complex. Similar to Myc and Max, Omomyc can inhibit the binding of WDR5 and KMT2A to DNA in the pulldown assay (Fig. 3E). These data suggest that Omomyc can potentially block various transcription factors and repressors from interacting with E boxes in promoters of Myc-regulated genes, confirming that Omomyc can “blunt” Myc interactions with E boxes (23).

**FIG 4:**
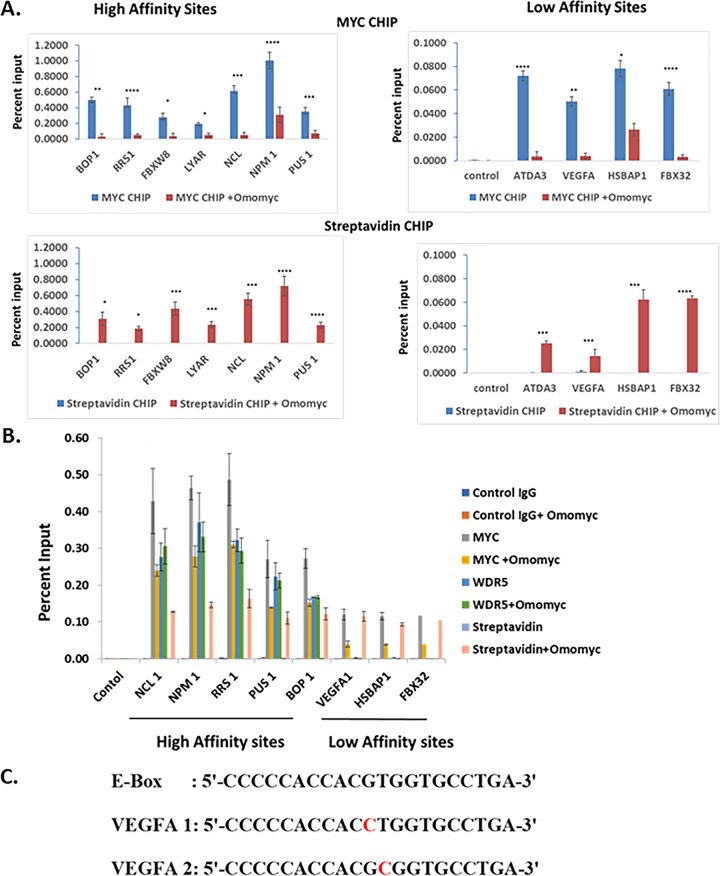
Omomyc can bind to DNA in cells (A) Chromatin immunoprecipitation assay demonstrating that Omomyc can displace Myc/Max heterodimers from binding to DNA and can bind to DNA directly. Immunoprecipitations were done with anti-Myc antibody, a control IgG, and streptavidin beads, which pull down biotinylated Omomyc. Genes assayed were the BOP1, RRS1, NCL1, FBXW8, LYAR, PUS1, ATDA3, VEGFA, FBX32, and HSBPA1 genes, along with a control region that does not contain any E box (data not shown). The statistical significance of data (*P* value) was calculated using two-tailed unpaired Student’s *t* test, which was done with GraphPad Prism. ****, *P* < 0.0001; ***, *P* < 0.005; **, *P* < 0.01; *, *P* < 0.05. (B) Chromatin immunoprecipitation assay to determine at which promoters WDR5 colocalizes with Myc and Omomyc. Antibodies to WDR5 and Myc and a control IgG were used along with streptavidin beads to immunoprecipitate DNA/protein complexes. Genes assayed were the NCL1, NPM1, RRS1, PUS1, BOP1, VEGFA-1, FBX32, and HSBAP1 genes, along with a control region that does not contain any E box. (C) Sequences of the consensus E box and E boxes found in the VEGFA promoter region. VEGFA E boxes 1 and 2 were used in the FP assay in Table 3 to determine the affinities of various dimers for each sequence.

We next tested whether Omomyc can bind DNA in the cell by performing a chromatin immunoprecipitation experiment with biotinylated Omomyc (Fig. 4A). HCT116 cells were treated with 2.5 or 10 μM Omomyc in the presence of ProteoJuice for 24 h and then fixed and lysed. The chromatin was then isolated, sheared, and immunoprecipitated with an antibody to Myc and either magnetic protein G beads or magnetic streptavidin beads. The immunoprecipitated chromatin was then subjected to RT-PCR with a series of primers to various promoters that contain high-affinity E boxes (NPM1, NCL1, BOP1, DBX8, LYAR, and RRS1) and low-affinity E boxes (ATDA3, vascular endothelial growth factor A [VEGFA], HSBAP1, and FBX32) (11, 23) as well as a control region in the genome. At both the high-affinity and low-affinity E box sequences, Omomyc displaced Myc from these promoters, as well as binding to the promoters itself (Fig. 4A and B).

The primary difference between the high-affinity and low-affinity sites was that the high-affinity sites tended to also bind WDR5 (Fig. 4B). The level of Myc at these promoters tends to mirror the amount of WDR5 present. In the E box DNA-coupled bead mass spectrometry assay, Omomyc added exogenously to a Ramos cell lysate could compete with both WDR5 and KMT2A for binding to E box DNA (Fig. 3E). These binding data suggest that Omomyc can block WDR5 and KMT2A from associating with E box DNA in the absence of Myc. At high-affinity promoters, Omomyc could not displace Myc any more than the amount of WDR5 present. At low-affinity promoters, there was no WDR5 present, and Omomyc could almost entirely displace Myc from these sites (Fig. 4B). This suggests that if an E box in a promoter is free from MYC or epigenetic factors that recruit MYC to the E box, Omomyc can bind and block binding of both Myc and epigenetic factors from the promoter.

One of these low-affinity sites is the VEGFA promoter, which contains two noncanonical E boxes (24) (Fig. 4C), in which a base central to the E box (CTCGTG) is replaced. A comparison of binding between various helix-loop-helix peptides shows that a Myc/Max dimer can bind to a consensus E box with relatively high affinity but binds the two nonconsensus E boxes in the VEGFA promoter approximately 4-fold more weakly (Table 3). Omomyc can bind to all three E boxes with approximately the same affinity, similar to a Myc/Myc dimer. A Max/Max homodimer or an ME-47 dimer, a hybrid protein consisting of a Max DNA binding domain fused to the helix-loop-helix dimerization domain of E47 (24, 25), binds to the consensus E box with an affinity similar to those of the Myc/Max heterodimer and Omomyc but fails to bind to the VEGFA E boxes (Table 3). This suggests that in cells, Omomyc can bind with a higher affinity to “weak” Myc promoters and can blunt the ability of Myc/Max heterodimers to bind these promoters and promote transcription.

**TABLE 3:** Affinities of bHLH peptides for consensus and nonconsensus E boxes^*a*^

Peptide	Affinity (nM)		
	E box	VEGFA-1	VEGFA-2
Omomyc	106.7	102.7	99.2
MYC/MYC	158.8	140.4	111.4
MAX/MAX	257.8	3,388.9	2,023
ME-47	117.4	1,076.9	3,108
MYC/MAX	71.4	397.3	451.2

a Affinity was measured by a fluorescence polarization (FP) assay as described in Materials and Methods.

### Omomyc is cell penetrant and localizes to the nucleolus in cells.

**FIG 5:**
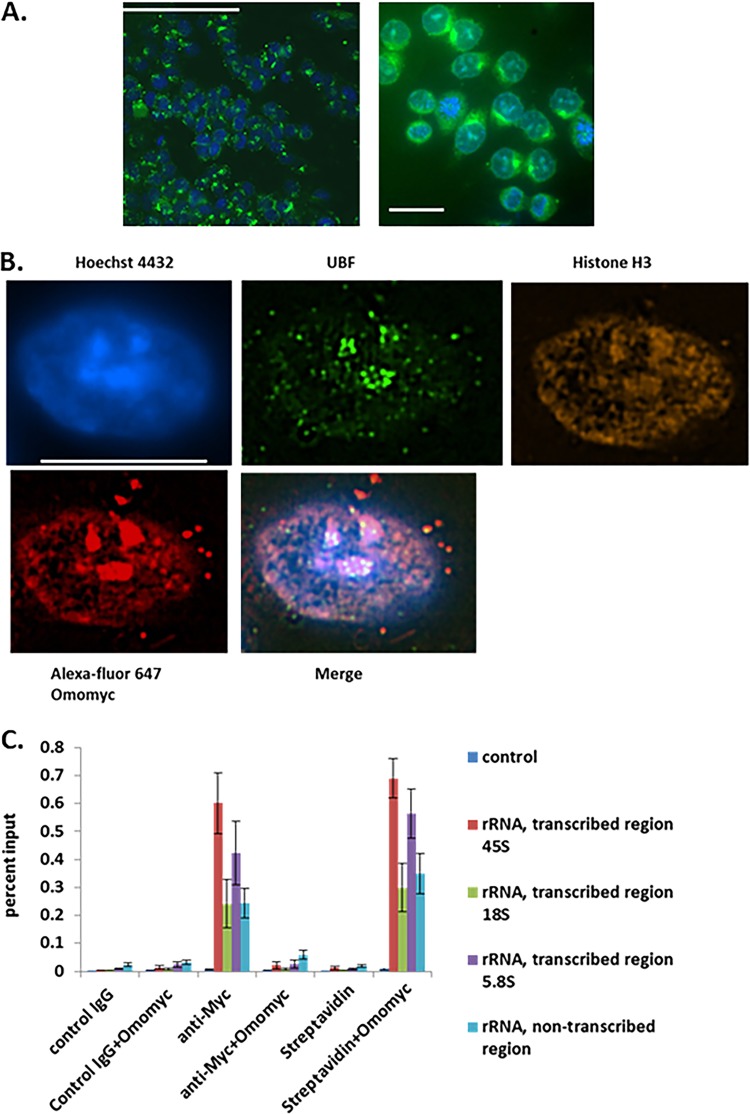
Omomyc is cell penetrant and localizes to the nucleolus. (A) Omomyc is cell penetrant. Fluorescein-labeled Omomyc was used to treat HCT116 cells for 24 h. Cells were fixed, stained with Hoechst 3342, and then visualized with a Molecular Dynamics ImageXpress high-content imager using 20× (left) and 60× (right) air objectives. Bars, 100 μm (left) and 10 μm (right). (B) Localization of Omomyc to the nucleolus. HCT116 cells were treated with 10 μM Alexa Fluor 647-Omomyc for 24 h, fixed, permeabilized, and stained with anti-UBF for the nucleolus, anti-histone H3 for DNA, and Hoechst 33342 for the nucleus. Cells were visualized with a Nikon SIM-E microscope at a ×100 magnification. Bar, 10 μm. (C) Omomyc binds to rDNA promoters and replaces Myc at these promoters. Cells were treated with 2.5 μM Omomyc plus ProteoJuice for 24 h and then fixed, nuclei were isolated, and chromatin was then sheared by sonication. Chromatin was then immunoprecipitated with antibodies to Myc, control IgG, and streptavidin. The chromatin was then eluted, treated with proteinase K, purified, and subjected to Q-PCR using Sybr green, using probes for transcribed rRNA regions for 45S rRNA, 18S rRNA, and 5.8S rRNA and an untranscribed rRNA region. All regions were previously shown to be bound by Myc and Mad1 (19, 28).

To determine if Omomyc can penetrate cells, we labeled Omomyc using maleimide-fluorescein isothiocyanate (FITC) via the free cysteine residue at amino acid 89. Using FITC-labeled Omomyc, we observed Omomyc uptake into cells (Fig. 5A), with the localization of Omomyc being perinuclear with some faint nuclear staining. The uptake of Omomyc into cells appears to be dependent upon active transport, since subjecting cells to a temperature of 4°C for 4 h or inhibiting ATP generation by low-dose treatment with sodium azide inhibits Omomyc uptake (data not shown), which is similar to the results demonstrated previously by Beaulieu et al. (26).

Using superresolution imaging techniques, we determined the subcellular localization of Omomyc. We utilized Alexa Fluor 647-labeled Omomyc to localize Omomyc in the cell. We found that Omomyc strongly localized to the nucleus, as evidenced by colocalization with Hoechst 33342 nuclear dye (Fig. 5B). In addition, Omomyc colocalized with UBF, a nucleolar transcription factor (28), by staining with an anti-UBF antibody. Besides colocalization with UBF, Omomyc was found to have punctate staining throughout the nucleus, correlating with anti-histone H3 staining, indicating that Omomyc was interacting with DNA in the nucleus (Fig. 5B). In addition, we stained cells with an anti-Max antibody and an anti-UBF antibody after treatment with 10 μM Alexa Fluor 647-Omomyc. Both Myc and the Myc-regulating protein Mad1 (Mxd1) have been reported to show strong binding to the nucleolus via immunofluorescence microscopy (19, 30) and to bind ribosomal DNA (rDNA) in the cell. Mad1 functions as an antagonist to Myc, blunting Myc’s transcriptional activation in the cell (13). To see if Omomyc was performing a similar function, we treated HCT116 cells with biotinylated Omomyc and then performed a CHIP assay using rDNA sequences that have been shown to bind both Myc and Mad1 (19, 30). Omomyc bound to the reported rDNA sequences quite strongly, while it failed to bind to the control DNA sequence lacking an E box (Fig. 5C). Given the binding and displacement of Myc from both DNA and Max, along with its nucleolar localization, Omomyc is potentially acting like Mad1, an antagonist of Myc-mediated transcription.

### Omomyc forms heterodimers with Max in the cell, which bind to promoter regions of Myc target genes.

**FIG 6:**
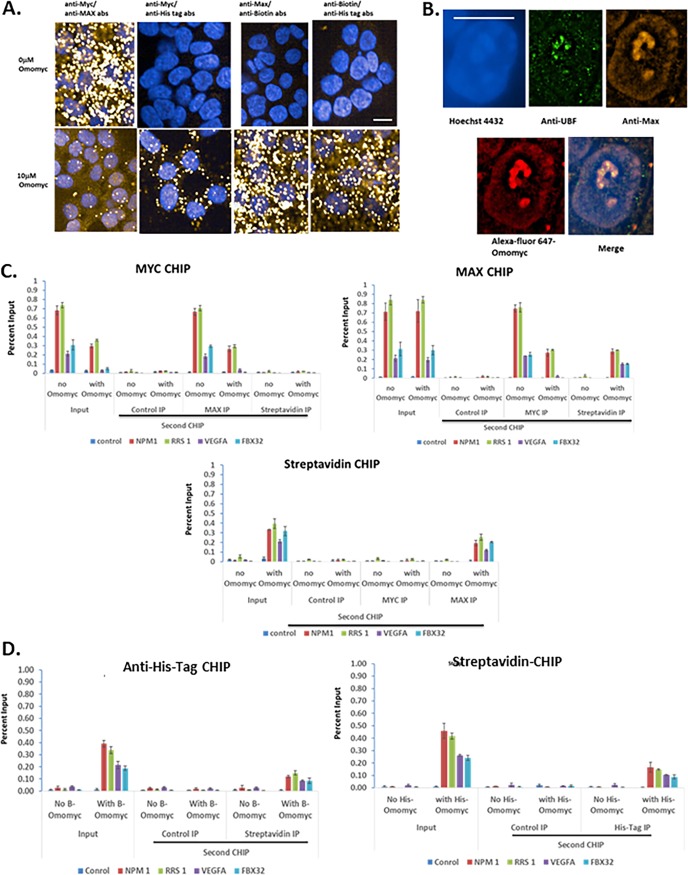
Interaction of differently labeled Omomyc, Myc, and Max both in the cell and at E boxes in promoters. (A) HCT116 cells were plated and then treated with a 10 μM mixture of biotinylated (5 μM) and 6×His-tagged (5 μM) Omomyc for 24 h. Cells were fixed in 3.9% formaldehyde, permeabilized, and then stained with antibodies to Myc, Max, biotin, or penta-His. The cells were then processed according to the instructions of the Sigma Duo-link kit. Cells were imaged on a Perkin-Elmer Opera Phenix high-content imager using a 40× water objective. Bar, 10 mm. (B) Omomyc colocalizes with Max in the nucleus and nucleolus. HCT116 cells were treated with 10 mM Alexa Fluor 647-Omomyc for 24 h, fixed, permeabilized, and stained with anti-UBF and Max antibodies as well as Hoechst 3342. Cells were imaged with a Nikon SIM-E microscope using a 100× objective. Bar, 10 mm. (C) ReCHIP assay for binding of Max and biotinylated Omomyc. HCT116 cells were treated with 2.5 μM Omomyc plus ProteoJuice for 24 h and then processed for chromatin immunoprecipitation by fixing the cells, isolating the nuclei, and shearing the chromatin. The initial chromatin immunoprecipitation was performed with antibodies to Myc and Max, control IgG, and streptavidin. The chromatin was eluted and then reimmunoprecipitated (ReCHIP) with the indicated antibodies. After the second chromatin immunoprecipitation, the eluted chromatin was treated with proteinase K, purified, and then subjected to Q-PCR with NPM1, RRS1, VEGFA-1, FBX32, and a control region that does not contain an E box. (D) ReCHIP assay demonstrating the interaction of Omomyc monomers on DNA. ReCHIP assays were performed as described above for panel B, using anti-6×His tag antibody and streptavidin for immunoprecipitation.

To provide further confirmation that Myc and Max can bind Omomyc and to localize the complexes in the cell, we performed a proximity ligation assay (PLA) in HCT116 cells. We treated the cells with a mixture of biotinylated Omomyc and His-tagged Omomyc (Fig. 6A). In the absence of Omomyc, only the interaction between Myc and Max is seen, mainly in the nucleus. Upon treatment with an equimolar mixture of biotinylated Omomyc and His-tagged Omomyc, there is a decrease in the interaction between Myc and Max. The Omomyc-Myc interaction occurs primarily in the cytoplasm, while the Omomyc-Max interaction occurs in both the nucleus and the cytoplasm (Fig. 6A). By superresolution microscopy, Max and Omomyc almost perfectly colocalize, especially in the nucleolus and along the periphery of the nuclear envelope, where transcription of Myc-activated genes occurs (31) (Fig. 6B).

Interestingly, we demonstrate that biotinylated Omomyc and His-tagged Omomyc can interact and are present in both the nucleus and the cytoplasm, suggesting that Omomyc dimer subunits can exchange in the cell (Fig. 6A). In total, the data indicate that Omomyc may exist as a dimer in solution, but in the cell, Omomyc appears to act as a monomer, exchanging subunits within the Omomyc homodimer, as well as forming heterodimers with Max and Myc.

Given the interaction between Omomyc and Max seen in the immunoprecipitation experiments and PLAs, we performed ReCHIP assays to determine whether Omomyc and Max were binding to the same promoter, which could potentially indicate that the Omomyc/Max heterodimer is functional in cells (Fig. 6C). HCT116 cells were treated with and without biotinylated Omomyc and then processed for chromatin immunoprecipitation. Omomyc decreased the amount of Myc present on various promoters. When a second immunoprecipitation was done with anti-Max antibody, the amount of Max pulled down was roughly similar to the amount of Myc on various promoters, while an additional streptavidin immunoprecipitation showed little or no binding of Omomyc to DNA that has Myc bound to it. Max can be immunoprecipitated from several promoters, with little change in the amount immunoprecipitated in the presence of Omomyc compared to untreated samples. When the second immunoprecipitation was done with a Myc antibody on two “strong” promoters, NPM1 and RRS, there was roughly a 60% reduction of Max and Myc binding to the promoter sequence in cells treated with Omomyc compared to untreated cells (Fig. 6C). On weaker promoters, VEGFA and FBX32, there was a complete loss of Myc binding to the same promoter as Max in the presence of Omomyc. With streptavidin in the second immunoprecipitation, streptavidin binding increased in the presence of Omomyc, suggesting that biotinylated Omomyc and Max are binding the same promoter sequence (Fig. 6C). As expected, streptavidin immunoprecipitation showed binding to promoters only in the presence of biotinylated Omomyc (Fig. 6C). The only significant binding when the second chromatin immunoprecipitation was done was with an antibody against Max, indicating that Omomyc and Max were bound at the same promoter.

To confirm the PLA results which demonstrated that Omomyc can homodimerize in the cell, we treated cells with equal molar amounts of 6×His-tagged Omomyc and biotinylated Omomyc and performed a ReCHIP assay (Fig. 6D). Both tagged versions of Omomyc bound to chromatin in the first CHIP. In the second CHIP, in which the other labeled Omomyc was immunoprecipitated, part of the first immunoprecipitated material was pulled down with the second immunoprecipitation, indicating that Omomyc dimers consisting of histidine-tagged and biotinylated Omomyc were bound together on chromatin (Fig. 6D). These results suggest that Omomyc can form dimers in the cell with either Max or other Omomyc monomers.

### Dimerization with Max occurs cotranslationally.

**FIG 7:**
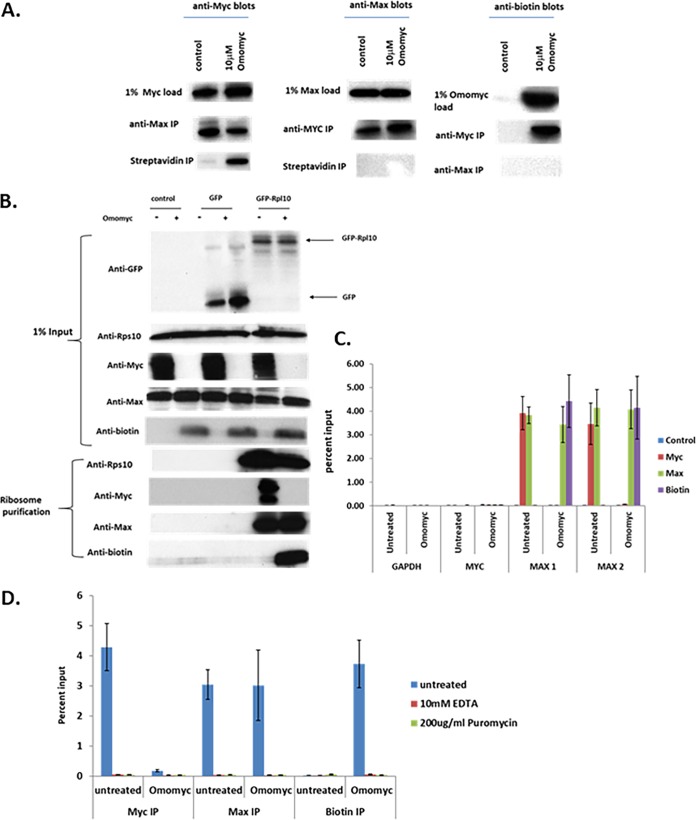
Myc/Max and Omomyc/Max heterodimers form cotranslationally. (A) Interaction of Omomyc with Myc and Max in HCT116 cell lysates. A total of 10 μM biotinylated Omomyc was added to an HCT116 cell lysate; incubated for 4 h; immunoprecipitated with either anti-Myc, anti-Max, or antibiotin antibodies; pulled down with magnetic beads; run on an SDS-PAGE gel; and then blotted with antibodies to Myc, Max, or biotin. (B) Myc, Omomyc, and Max associate with translating ribosomes. GFP and GFP-Rpl10 plasmids were transfected into HCT116 cells for 24 h and then treated with ProteoJuice with and without 10 μM Omomyc for 6 h. The cells were lysed, and ribosomes were affinity purified with anti-GFP antibody. Purified ribosome complexes were washed and then blotted for the presence of Myc, Max, and biotinylated Omomyc. (C) RNA immunoprecipitation (RIP) assay demonstrating that Myc, Max, and Omomyc can bind MAX RNA. HCT116 cells were plated overnight, treated with ProteoJuice with and without 10 μM Omomyc for 6 h, lysed, and then immunoprecipitated with antibodies to Myc, Max, and biotin. After immunoprecipitation, RNA was isolated from the immunoprecipitates, cDNA was made, and RT-PCR was performed for the primer/probe sets indicated. (D) Myc, Max, and Omomyc interact with MAX RNA in a complex involving ribosomes. HCT116 cells were treated as described above for panel B. Two hours before lysis, 200 μg/ml puromycin was added to cells to inhibit protein translation. At 6 h, cells were lysed, and some lysates had 10 mM EDTA added to dissociate polysomes. Samples were treated as described above for panel B, and RT-PCR was performed with the primer/probe sets indicated.

To determine whether Omomyc can interact with either Myc or Max in a cell lysate, we added 10 μM biotinylated Omomyc to HCT116 cell lysates and, after a 4-h incubation, immunoprecipitated Myc, Max, and biotinylated Omomyc from the lysates (Fig. 7A). Myc and Max were immunoprecipitated with each other, although in the presence of Omomyc, there was a decrease in the amount of Max that was immunoprecipitated with Myc. Myc was also immunoprecipitated with biotinylated Omomyc, while Max did not immunoprecipitate with Omomyc. These results suggest that the interaction between Myc and Max may be cotranslational, and once Max is in a complex with another binding protein, it is unavailable to bind Omomyc.

Given the possibility that Myc and Max could associate cotranslationally, we performed a modified translating ribosome affinity purification (TRAP) assay to test whether Myc, Max, or Omomyc could associate with translating ribosomes. We transfected an expression plasmid encoding either green fluorescent protein (GFP) or GFP-RPL10, a 60S subunit ribosomal protein, into cells and then subsequently treated the cells with or without 10 μM biotinylated Omomyc for 6 h. We then lysed the cells, isolated the translating ribosomes using an antibody to GFP to pull down the tagged GFP-Rpl10 protein, and immunoblotted for RPS10, a 40S ribosomal subunit protein, Myc, Max, and Omomyc to determine which proteins were in the protein complex with Rpl10 (Fig. 7B). In cells transfected with the GFP-tagged Rpl10 expression plasmid, both Rps10 and Max were complexed with the ribosomes in the presence and absence of Omomyc. In untreated cells, Myc associated with the translating ribosome. When cells were treated with Omomyc, Myc association with ribosomes disappeared, replaced by Omomyc association with the ribosomes (Fig. 7B). These TRAP results indicate that Myc and Max can associate with translating ribosomes in the absence of Omomyc and that in the presence of Omomyc, Myc ribosomal association is inhibited. Therefore, Myc, Max, and Omomyc may associate with Max cotranslationally.

To further confirm the potential cotranslational association between Myc, Max, and Omomyc, we then performed an RNA immunoprecipitation (RIP) assay to test whether Myc, Max, and Omomyc could associate with RNAs of potential binding partners, which is seen with other proteins that cotranslationally associate (32, 33). HCT116 cells were treated with ProteoJuice transfection reagent and 10 μM biotinylated Omomyc for 4 h, lysed, and immunoprecipitated with antibodies to Myc, Max, biotin, or a control rabbit IgG overnight. The immunoprecipitates were then washed, the RNA was eluted, cDNA was synthesized, and RT-PCR was performed on the samples (Fig. 7C). None of the immunoprecipitations pulled down RNA for Gapdh (glyceraldehyde-3-phosphate dehydrogenase) or Myc, while in untreated cells, both Myc and Max associated with Max RNA, as demonstrated by the amplification of two different Max probes (MAX 1 and MAX 2) in the assay. In Omomyc-treated cells, biotinylated Omomyc and Max bound Max RNA, while Myc binding to Max RNA was ablated by Omomyc (Fig. 7C). Cells were then treated with and without 10 μM Omomyc, and ribosomes were then disrupted by either treatment of cells with 200 μg/ml puromycin or lysis of cells and incubation with 10 mM EDTA. Dissociation of the large and small ribosomal subunits abolishes all binding of Myc, Max, and Omomyc to MAX RNA (Fig. 7D). Therefore, binding of MYC and Omomyc to Max RNA occurs in a complex with intact ribosomes, indicating that dimerization of MAX to Omomyc and MYC is cotranslational.

### *In vivo*, Omomyc rapidly distributes to tissues.

**FIG 8:**
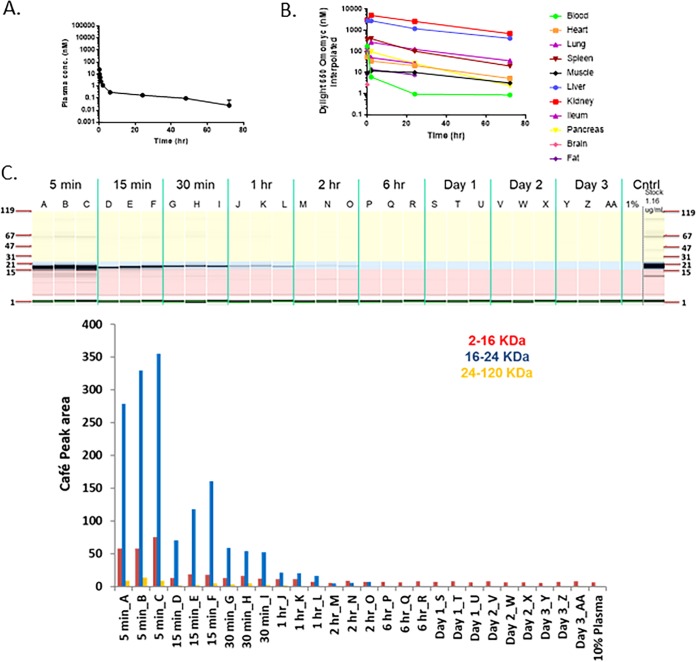

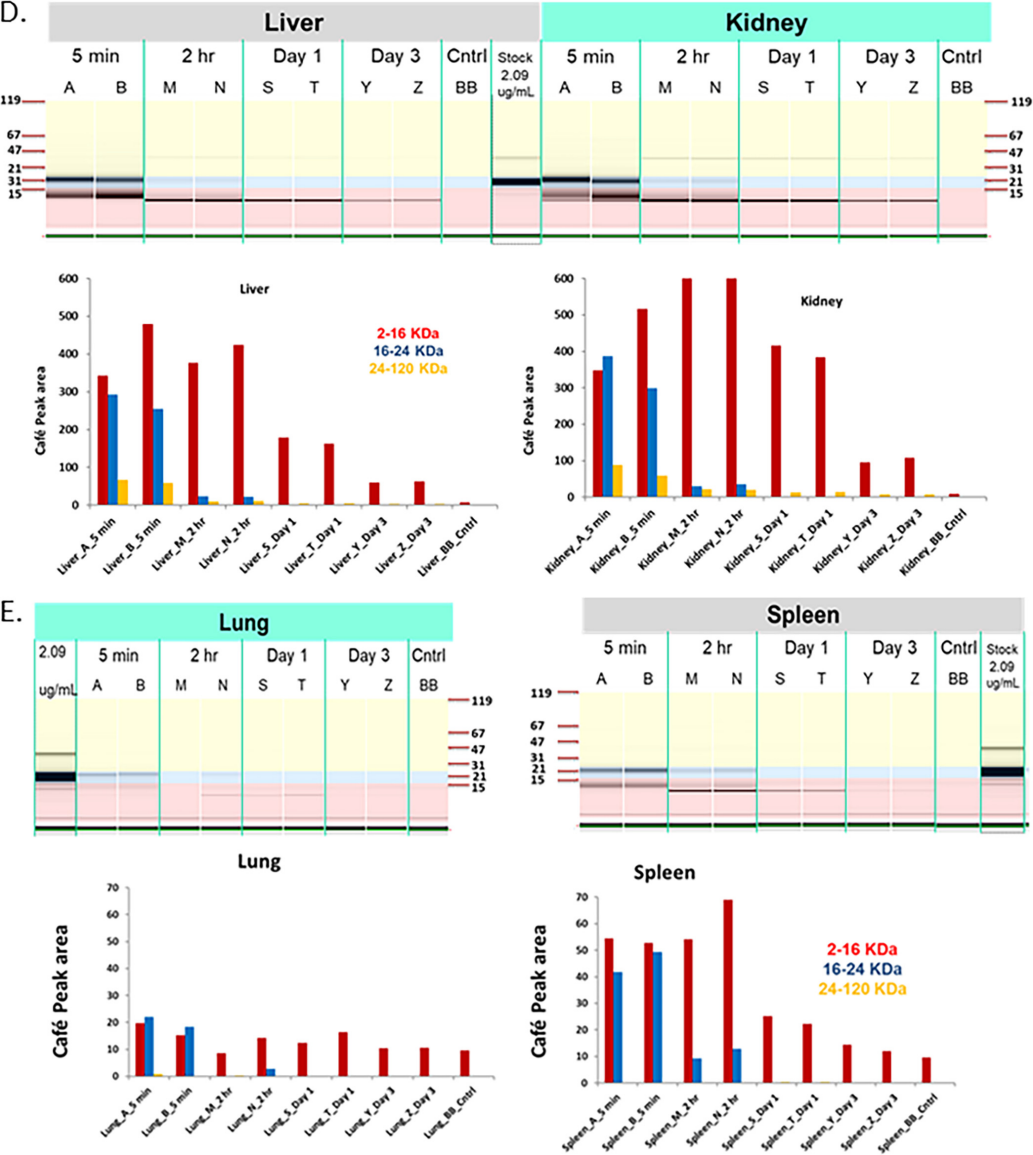
Omomyc displays poor pharmacodynamic properties. (A) Plasma concentration of DyLight 650-labeled Omomyc after administration to mice. A total of 5.22 mg/kg DyLight 650-Omomyc was administered to mice in a single i.v. dose. Terminal plasma samples were collected over time, and the amount of the compound was determined. (B) Tissue distribution of DyLight 650-Omomyc. Tissue samples were collected at the indicated times and analyzed for the presence of the compound. (C) CAFÉ analysis of plasma from non-tumor-bearing mice treated with Omomyc. Shown are examples of capillary electrophoresis and quantitation of the area under the curve for the peaks from the electropherograms for plasma from mice treated with DyLight 650-Omomyc. (D) CAFÉ analysis of tissues from non-tumor-bearing mice treated with Omomyc. Shown are examples of capillary electrophoresis and quantitation of the area under the curve for the peaks from the electropherograms for mouse plasma liver, kidney, spleen, and lung tissue from mice treated with DyLight 650-Omomyc.

Recent results from Beaulieu et al. (26) suggest that Omomyc is potentially an effective antitumor agent in animal models. To determine if Omomyc could be used in *in vivo* experiments, we first characterized the stability of Omomyc in mouse plasma. We found that the Omomyc monomer rapidly disappeared in mouse plasma, possibly due to the free cysteine at residue 89 in Omomyc binding to plasma proteins and/or binding to itself. We then made a covalent dimer, linking two Omomyc monomers via a disulfide bond at cysteine 89. This covalent dimer demonstrated much better stability than the monomer in mouse plasma (data not shown). To assess stability, pharmacokinetics, and disposition, the DyLight 650 fluorescently labeled Omomyc dimer was dosed intravenously (i.v.) in non-tumor-bearing BALB/c mice at 5.22 mg/kg of body weight (Fig. 8A to C). The Omomyc dimer showed biphasic kinetics in plasma, where its levels rapidly declined in mouse plasma in the initial distribution phase, followed by a slow terminal elimination phase to levels that were >180-fold below the EC_50_ needed for *in vitro* efficacy or a pharmacodynamic (PD) effect based on a tumor cell model (Fig. 8A). We found that Omomyc distributes mainly to the liver and kidneys, with only small amounts reaching other tissues, primarily spleen and ileum (Fig. 7B), where it is degraded from the tissue quickly as determined by CAFÉ (capillary fluorescent electrophoresis) analysis (Fig. 8C and D). Overall, Omomyc declines rapidly in mouse plasma due to a fast distribution. The sustained low plasma level during the terminal elimination phase and fast catabolism in tissues could limit its use *in vivo* in preclinical models.

## DISCUSSION

When Omomyc is expressed in cells, it has been shown to bind to DNA and affect cell growth (20–23). Here, we show similar effects on cells treated with either recombinant or synthetic Omomyc protein. Omomyc was designed to bind to Myc and inhibit the Myc/Max interaction (20, 21). Although derived from the bHLH domain of Myc, Omomyc has several residues mutated to the residues found in the bHLH domain of Max to improve its binding to Myc (20). Further studies indicated that Omomyc can bind proteins in the Myc network, such as Max and Miz1, but it fails to bind Mad1 (21). In addition, Omomyc can alter the level of histone acetylation in the cell, suggesting that it prevents Myc/Max heterodimers from binding to DNA, preventing them from interacting with histone acetylators (21). A second mechanism of action has been proposed by Jung et al. (23), in which Omomyc binds to E boxes in the promoter sequence and “blunts” the ability of Myc/Max heterodimers to bind DNA We have confirmed that Omomyc can bind to DNA *in vitro*. We have demonstrated by chromatin immunoprecipitation (CHIP) that exogenously applied Omomyc can act like ectopically expressed Omomyc and bind to promoter E boxes in the cell. Much like ectopically expressed Omomyc, recombinant or synthetic Omomyc protein can bind to both “strong” and “weak” E boxes. In the case of strong E boxes, the amount of binding is dependent on the level of WDR5 bound at the promoter. The central region of Myc has been shown to bind to WDR5, which helps stabilize Myc/Max heterodimer binding to E boxes (10, 11). Binding to WDR5 also facilitates Myc interaction with other components of the MLL complex to promote histone H3K4 methylation and increase Myc-mediated transcription (34). In the absence of WDR5, Omomyc is easily able to displace unstable Myc/Max heterodimers. *In vitro*, Omomyc can bind to weak noncanonical E boxes such as those in the VEGFA promoter with a much higher affinity than a Myc/Max bHLH peptide or a Max/Max bHLH peptide homodimer.

**FIG 9:**
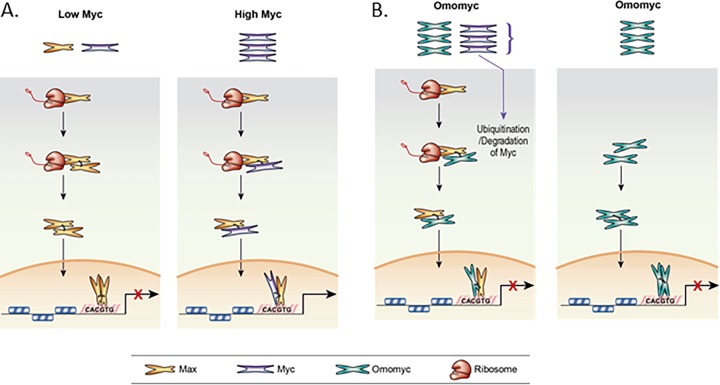
Models for Max cotranslational association with Myc and Omomyc inhibition of MYC transcriptional activity. (A) Cotranslational binding of Myc to Max when Myc levels are low (normal cells) and when Myc levels are high (transformed cells). (B) Two models of how Omomyc inhibits Myc-mediated transcriptional activity in the cell. (Left) Cotranslational binding of Omomyc to Max, blocking Myc binding to Max, leading to Myc degradation. (Right) Direct binding of Omomyc to E boxes in promoters of Myc-regulated genes as proposed previously by Jung et al. (23).

Our data demonstrate that the mechanism of Omomyc inhibition of Myc appears to be a combination of the mechanism proposed by Jung et al., where Omomyc dimers can bind DNA directly (23), and a mechanism more akin to the cellular antagonist of Myc, Mad1 (Mxd1) (13–15) (Fig. 9). Recent data indicate that Omomyc in solution forms stable homodimers or heterodimers with Max, further supporting a potential dual mechanism (26). When cells are treated with Omomyc, the Myc levels decrease, thus decreasing the level of Myc/Max heterodimers in the cell. Inhibition of the proteasome stabilizes the level of Myc in the cell and the level of Myc/Max heterodimers. In the presence of cycloheximide, Omomyc still bound Max and increased the amount of Max present. There was a decrease in the levels of Myc over time and a significant decrease in Myc/Max or Myc/Omomyc binding in the presence of cycloheximide, similar to what has been previously reported (35).

After treating cell lysates with Omomyc, we found that Omomyc bound to Myc in the cell lysates but not Max, suggesting that Max is present not as a monomer in the cell lysate but in a homodimer or in a heterodimer with Myc or another interacting protein. The binding of Myc to Omomyc in the cell lysate indicates that there was excess Myc monomer present and available to bind to Omomyc, unlike Max. This suggests that Max could form homodimers or heterodimers cotranslationally, in a mechanism that has been shown previously for Set1 (32, 33) (Fig. 9A). In a TRAP assay, Myc, Max, and Omomyc associate with translating ribosomes, indicating the potential for cotranslational interaction with Max protein. In the absence of Omomyc, Max and Myc are present in association with the ribosomes. Upon Omomyc treatment, Myc association with the ribosome disappears, being replaced by Omomyc. This is further confirmed by our RIP assay data, where Myc and Max interact with Max RNA in untreated cells. When cells are treated with Omomyc, Myc binding to Max RNA is significantly decreased, while the binding of Omomyc to Max RNA appears to be similar to the levels of Myc/Max RNA binding seen in untreated cells. Treatment with either puromycin or incubation of lysates with 10 mM EDTA to dissociate ribosomes completely reduces binding to Max RNA, indicating that binding of Myc, Max, or Omomyc to Max RNA is dependent upon the presence of intact ribosomes, a requirement for cotranslational binding of proteins (32, 33, 36).

In normal cells, the Myc level is relatively low, and when Max is translated, Max interacts with either available Myc monomers or Max monomers. With low Myc levels, the Max homodimers predominate, and transcription of Myc-controlled genes is repressed by the Max homodimers. Myc stabilization or amplification increases Myc levels and unbalances the ratio of Myc to Max (Fig. 9A). This leads to increases in the level of Myc/Max heterodimers forming cotranslationally, leading to increases in Myc-mediated transcription of Myc target genes and the availability of components for ribosomal biogenesis (19, 31, 37, 38).

A cotranslational model for Myc/Max and Max/Max associations is different from the current model, in which the association of both heterodimers of Myc and Max as well as homodimers of Max form via the initial binding of one monomer to DNA, with subsequent cooperative binding of the second monomer to form the dimer bound to DNA. Although seen *in vitro* (39), such a mechanism has not been demonstrated *in vivo*. A similar cotranslational mechanism of association has been demonstrated by Nicholls et al. (40) for p53, where p53 dimers form initially cotranslationally and subsequent tetramers are formed soon after the formation of dimers. Cotranslational formation of Myc/Max dimers may help protect Myc from degradation and help mediate translocation into the nucleus, since the free Myc monomer appears to be unstable in the cell.

Omomyc treatment appears to rebalance the Myc/Max ratio by binding both Myc and Max proteins and preventing their interaction. Omomyc can function like Mad1, where it preferentially forms dimers with Max and represses Myc-mediated transcription (Fig. 9B) (13–18). Like Mad1, Omomyc localizes to the nucleolus and binds to rDNA promoters, which may lead to a decrease in the components necessary for ribosomal biogenesis, affecting cell growth and proliferation (18). Due to its ability to bind to Myc, Omomyc can bind to excess Myc monomer in the cytoplasm and prevent it from entering the nucleus. The cytoplasmic Omomyc/Myc heterodimer would then be ubiquitinated and degraded, much like the free Myc monomer in the cell. In PLAs, Omomyc preferentially binds to Max, and given the size of the heterodimer, it could easily transit into the nucleus and function like a Mad1/Max heterodimer or a Max homodimer to inhibit Myc-mediated transcription. Similarly, Omomyc homodimers can also freely transit into the nucleus and inhibit Myc-mediated transcription (Fig. 9B). Both Omomyc/Max heterodimers and Omomyc homodimers can bind to DNA and displace Myc/Max heterodimers, especially at weak promoters, where the Myc/Max heterodimer is not stabilized by WDR5 (Fig. 9B) (10).

Similar to Mad1, Omomyc can antagonize the formation of Myc/Max heterodimers and disrupt Myc-mediated transcription. Unlike Mad1, Omomyc can potentially interact with Miz1. Like the Myc/Miz1 interaction, Omomyc/Miz1 may mediate transcriptional repression of the cell cycle inhibitors CDKN2B and CDKN1A, allowing for cell cycle progression and growth and potentially limiting the effectiveness of Omomyc (21, 41–43). Savino et al. suggested that modification of Omomyc to hamper Miz1 binding may help in both ablating the *trans*-repression activity seen with Omomyc treatment and increasing Omomyc efficacy (21).

Recent studies have indicated that compounds that can stabilize Max homodimer formation can lead to a decrease in the Myc protein level as well as a decrease in Myc-mediated transcription in a manner similar to that of Omomyc (44). Such small molecules may bind to the nascent Max polypeptide during translation, prevent the association of Max with Myc, and promote Max homodimerization. It will be interesting to see if such compounds also affect Max heterodimerization with other Max binding partners besides Myc.

Similar to the results of Beaulieu et al. (26), our data indicate that Omomyc persisted for 70 h in plasma. In the present study, with multiple early sampling time points, we were able to fully capture the Omomyc PK profile. Our results suggest that Omomyc displays a biphasic decline in plasma, with an initial sharply declining distribution phase followed by a slow terminal phase. As a result, Omomyc concentrations rapidly fell below the EC_50_ to be effective in a tumor cell line model and were sustained at a low level in the terminal phase. The sharp decline of Omomyc in plasma was also evidenced by rapid tissue uptake, primarily to the liver and kidneys, followed by a fast catabolism of Omomyc in tissue as assessed by CAFÉ analysis.

Collectively, our data demonstrate that Omomyc is a cell-penetrant MYC inhibitor with a dual mechanism of action. Omomyc binds to DNA as a homodimer, preventing Myc/Max interaction with DNA, and forms Omomyc/Max heterodimers that occurs cotranslationally to prevent the formation of Myc/Max heterodimers to repress Myc-mediated transcription. Although it is cell penetrant, Omomyc’s pharmacokinetic properties could limit its use *in vivo* in preclinical models.

## MATERIALS AND METHODS

### Cell culture and transfection.

HCT116, Ramos, A2780, Raji, Daudi, CA46, Jiyoye, KM-H2, L-428, HDLM-2, HD-MY-Z, and MSTO-211H cells were obtained from the ATCC (Manassas, VA). BJAB cells were obtained from the DKMZ (Germany). RPMI 1640 medium and Dulbecco’s modified Eagle’s medium (DMEM) were obtained from Thermo-Fisher (Grand Island, NY), and fetal bovine serum (FBS) was obtained from Sigma (St. Louis, MO).

### Expression and purification of Omomyc.

Omomyc was cloned into a pET47b (EMD-Sigma, Bedford, MA) E. coli expression vector and transformed into BL21A1 one-shot cells (Thermo-Fisher, Carlsbad, CA). Bacterial colonies were picked and grown in 2XYT medium (Teknova, Hollister, CA) at 37°C until the optical density at 600 nm (OD_600_) reached 0.8. The culture was induced by the addition of 1 mM isopropyl-β-d-thiogalactopyranoside (IPTG) (Sigma, St. Louis, MO), and the culture continued to grow at 37°C for 16 h before being harvested by centrifugation. The harvested pellet was resuspended in a solution containing 50 mM KH_2_PO_4_, 0.7 M NaCl, and 5 mM MgCl_2_ plus a protease inhibitor cocktail (EMD-Sigma). In addition, 150 μl of Triton X-100 (Sigma) per g of pellet was added to reduce viscosity. The suspension was sonicated on ice using a Branson (Danbury, CT) sonicator six times for 15 s at power setting 40. DNA was eliminated by adding 100 μl per g of pellet of a DNase I solution containing bovine pancreatic DNase (EMD-Sigma), 50 mM Na-acetate (pH 5.2), 1 mM CaCl_2_, and 50% glycerol. The suspension was incubated at 37°C for 1 h with agitation at 100 rpm and then centrifuged. Inclusion bodies were resuspended in a solution containing 50 mM Na-acetate (pH 5.2), 6 M urea, 0.5 M guanidine-HCl, 25 mM dithiothreitol (DTT), and a protease inhibitor cocktail by vortexing. The suspension was diluted 1:1 with 2 M urea, followed by centrifugation at 20,000 rpm for 30 min. The collected supernatant was then loaded onto two preequilibrated (50 mM Na-acetate, pH 5.2) 5-ml SPFF columns (GE Bioscience, Westborough, MA) in tandem, attached to an Äkta Explorer fast protein liquid chromatography (FPLC) instrument (GE Bioscience). The columns were washed with a solution containing 50 mM Na-acetate (pH 5.2) plus 30 mM NaCl. Omomyc was eluted from the columns with a 10 to 100% linear gradient using a solution containing 50 mM Na-acetate (pH 5.2) plus 3 M NaCl. Pooled fractions containing Omomyc were determined by sodium dodecyl sulfate-polyacrylamide gel electrophoresis (SDS-PAGE), concentrated, and loaded onto a GE Bioscience Superdex-75 26/600 column for further polishing with a solution containing 1× Dulbecco's phosphate-buffered saline (GE Bioscience) (pH 7.2) plus 10% glycerol as the final buffer. Fractions containing pure Omomyc were determined, pooled, and concentrated for analytical analysis before use or stored at −80°C.

### Proliferation assays.

Proliferation assays were performed by seeding cells at 1,000 to 2,000 cells/well in a 96-well plate for HCT116 cells and at 5,000 cells/well for lymphoma cells. Cells were then treated with a titration of Omomyc in phosphate-buffered saline (PBS) (Thermo-Fisher, Grand Island, NY) with 2 mM DTT (Pierce) for 72 h. At 72 h, each well received an equal volume of CellTiter Glo (Promega, Madison, WI), and each plate was read on a Wallac 1420 multilabel counter. Proliferation was calculated as the percentage of untreated cells and graphed with Prism (GraphPad, San Diego, CA), using nonlinear regression and a sigmoidal dose-response formula.

### Cell penetration assay.

HCT116 cells were plated in Greiner glass-bottom plates that were treated with poly-d-lysine prior to plating of cells. The cells were treated with 20 μM FITC-labeled Omomyc for 4 h, with one plate incubated at 37°C and another plate shifted to 4°C. Cells were then fixed with 4% formaldehyde, washed in PBS, treated with 0.01% trypan blue (Thermo-Fisher) for 5 min, and then rinsed in PBS. Plates were visualized at a ×20 magnification on an ImageXpress high-content imager from Molecular Devices.

### RNA-Seq and RT-PCR.

For RNA-Seq, HCT116 cells were treated with and without 2.5 or 10 μM Omomyc with ProteoJuice for 6 or 24 h, and RNA was then isolated with an RNeasy kit (Qiagen). For RT-PCR, HCT116 cells were treated for 24 h with or without 10 mM Omomyc in the presence of ProteoJuice. At 24 h, RNA was isolated by using an RNeasy kit as described above, and cDNA was generated with Superscript Vilo (Thermo-Fisher, Waltham, MA). After generation, cDNA was diluted 1:30, and RT-PCR was performed with TaqMan RT-PCR master mix (Thermo-Fisher, Waltham, MA) using primer/probe sets for ASNS, ID3, EGR2, and CD274 (Thermo-Fisher) (see Table SI in the supplemental material). Fold expression was calculated by the ΔΔ*C_T_* method, with the means and standard deviations (SD) calculated and graphed in Excel.

### Immunoprecipitation and Western blotting.

Immunoprecipitations were performed after HCT116 cells were lysed with cell lysis buffer (Cell Signaling Technology, Danvers, MA) and then clarified by centrifugation at 13,000 rpm for 10 min to remove cell debris. Antibodies (indicated in Table SI) were added and incubated for the indicated times, and antibodies were then precipitated with protein G magnetic beads. Samples with biotinylated Omomyc were precipitated with streptavidin magnetic beads (Thermo-Fisher, Carlsbad, CA). Magnetic beads were then washed 5 times with cell lysis buffer and radioimmunoprecipitation assay (RIPA) buffer (Cell Signaling Technology, Danvers, MA). Beads were resuspended in 1× sample buffer (Thermo-Fisher, Carlsbad, CA). Samples were then subjected to Western blotting by running samples on a 4 to 20% polyacrylamide gel (Thermo-Fisher, Carlsbad, CA) and then transferring them to a polyvinylidene difluoride (PVDF) membrane using an I-blot apparatus (Thermo-Fisher, Carlsbad, CA). Membranes were then probed with the appropriate primary antibody, followed by a secondary antibody, either goat anti-rabbit antibody–horseradish peroxidase (HRP) or goat anti-mouse antibody–HRP (Cell Signaling Technology, Danvers, MA). Blots were developed with ECL developing solution (GE Bioscience, Westborough, MA) and exposed to X-ray film (GE Bioscience, Westborough, MA).

### ProteoJuice treatment.

To increase Omomyc penetration into cells, ProteoJuice protein transfection reagent (EMD-Millipore, Burlington, MA) was used. ProteoJuice was first vortexed for mixing and then combined with Omomyc and Opti-MEM for 20 min. At 20 min, additional Opti-MEM was added, and a minimal volume of the transfection cocktail was applied to just cover the cells; for incubations longer than 4 h, media with 10% FBS were added to prolong cell survival.

### FITC labeling and imaging of Omomyc.

Omomyc was labeled with maleimide-FITC (Pierce/Thermo-Fisher, Waltham, MA), with excess label being removed using a G25 spin column (GE Bioscience, Westborough, MA). Cells were treated with 20 μM FITC-labeled Omomyc for 6 h and fixed with 4% formaldehyde (Sigma, St. Louis, MO) for 15 min. Cells were then rinsed with PBS (Thermo-Fisher, Grand Island, NY), stained with 0.2% Hoechst 33342 nuclear dye for 5 min, rinsed with PBS again, and finally visualized at ×20 and ×60 magnifications using a Molecular Devices (Sunnyvale, CA) ImageXpress high-content imager.

### Synthesis of the Omomyc monomer.

The protein sequence was assembled by solid-phase synthesis on a Microwave Liberty Blue synthesizer (CEM, Matthews, NC). The N and C termini were blocked using an N-acetyl and a C-carboxamide, respectively. Synthesis was started using either 100 μmol of PEG-PS ProTide resin LL, 100 to 200 mesh, with loading equal to 0.19 mmol/g (CEM). Each amino acid was coupled in 9-fold excess as a 0.2 M solution in dimethylformamide (DMF), which was activated using a 9-fold excess of 0.5 M diisopropylcarbodiimide (DIC) and 1 M Oxyma in DMF. Single and double couplings were performed at 90°C with 5-min coupling times, except for 9-fluorenylmethoxy carbonyl (Fmoc)-His(Trt)–OH, which was coupled at 50°C. Underlined amino acids in the sequence were double coupled. Aspartic acid was coupled as Fmoc-Asp(OBno)–OH. For coupling cycles following Asp(OBno)-OH, the Fmoc protecting group was deprotected at room temperature (RT) to minimize base-promoted aspartimide formation.

The following protected natural amino acids were used: Fmoc-Ala–OH, Fmoc-Arg(Pbf)–OH, Fmoc-Asn(Trt)–OH, Fmoc-Asp(OBno)–OH, Fmoc-Cys(Trt)–OH, Fmoc-Gln(Trt)–OH, Fmoc-Glu(OtBu)–OH, Fmoc-His(Trt)–OH, Fmoc-Ile–OH, Fmoc-Leu–OH, Fmoc-Lys(Boc)–OH, Fmoc-Phe–OH, Fmoc-Pro–OH, Fmoc-Ser(t-Bu)–OH, Fmoc-Thr(t-Bu)–OH, Fmoc-Tyr(t-Bu)–OH, and Fmoc-Val–OH.

Release and deprotection of Omomyc from its solid support were achieved by stirring the peptidyl resin in TFA (trifluoroacetic acid)-water-TIS (triisopropylsilane)-phenol (87.5:5:2.5:5, vol/vol/vol/wt; 15 ml/g of peptidyl resin) for 2 to 3 h at room temperature. After removal of the spent resin by filtration and rinsing with a trifluoroacetic acid (TFA) cleavage solution, the combined TFA filtrates were concentrated under reduced pressure. The crude protein product was precipitated and washed with diethyl ether. It was then purified to >90% purity using preparative mass-triggered high-performance liquid chromatography (HPLC) on a C_8_ column (Reprosil 200A, 5 μm), C_4_ reverse-phase columns, and linear gradients of acetonitrile (ACN) in water, both buffered with 0.1% TFA. The HPLC fractions containing a pure protein product were pooled and lyophilized to yield Omomyc as a white solid in TFA salt form. The desired molecular weight (MW) of the purified protein was confirmed by electrospray ionization-mass spectrometry (ESI-MS) analysis.

Conversion of the TFA to the acetate salt form of the protein was performed using a HiTrap Q HP strong-anion-exchange column (5 ml; GE Healthcare).

### Synthesis of the biotin-tagged Omomyc monomer.

Biotin-tagged Omomyc was prepared by conjugation with maleimide-PEG_2_-biotin as follows. Omomyc acetate (45.5 mg; 4.11 μmol), Tris(2-carboxyethyl)phosphine hydrochloride (TCEP) (1.649 mg; 5.75 μmol), and EZ-Link maleimide-PEG_2_-biotin (8.10 mg; 15 μmol) (Thermo-Fisher Scientific) were dissolved in degassed water (2.0 ml) in a 4-ml vial with a pressure release septum cap. The vial was purged with nitrogen for a few minutes, and the resulting clear solution was stirred gently for 2 h under nitrogen.

The reaction mixture was diluted to 12 ml with water, and the solution volume was reduced to 1.5 ml by centrifugation using an Amicon Ultra-15 3K centrifugal filter device (Millipore). This procedure was repeated five times. The final remaining 1.5 ml of the product solution was transferred to a vial and lyophilized to yield 37.4 mg (89%) of a >90% pure product as a white solid. The MW of the correct product was confirmed by ESI-MS analysis.

### Synthesis of the His-tagged Omomyc monomer.

His-tagged Omomyc was prepared as described above for biotin-tagged Omomyc, except that maleimide–hexa-His was used for conjugation with the Cys89 residue of Omomyc. Maleimide–hexa-His was prepared using standard solid-phase synthesis methods.

### Synthesis of the Alexa Fluor 647-tagged Omomyc monomer.

Alexa Fluor 647-tagged Omomyc was prepared as described above for biotin-tagged Omomyc, except that Alexa Fluor 647 C2 maleimide (Thermo-Fisher Scientific) was used for conjugation with the Cys89 residue of Omomyc.

### Synthesis of the Omomyc covalent dimer.

The purified Omomyc monomer (4.243 g; 0.324 mmol) was dimerized via disulfide formation by stirring it in a solution of water (48.0 ml) and 10× Tris-buffered saline (TBS) (pH 7.4) (48 ml) adjusted to pH 7.8 by the addition of 1.2 ml of 5 M NaOH, followed by the addition of dimethyl sulfoxide (DMSO) (21.0 ml). Liquid chromatography-mass spectrometry (LC-MS) analysis showed the reaction to be complete after 16 h at room temperature. The reaction mixture was freeze-dried to a solid, which was purified to >90% purity using preparative mass-triggered HPLC on a C_18_ reverse-phase column and linear gradients of acetonitrile in water, both buffered with 0.1% TFA. The HPLC fractions containing a pure protein product were pooled and lyophilized to yield the Omomyc dimer as a white solid in TFA salt form. The desired product mass was confirmed by ESI-MS analysis.

Conversion of the TFA to the acetate salt form of the protein was performed using a custom column packed with Fast Flow Q Sepharose gel (42 ml; 0.2 mmol/ml [acetate form]) (strong anion exchange; GE Healthcare). A solution of the protein dimer (0.5 to 1.0 g) in water (12 to 25 ml) was loaded onto the column, and the protein was eluted using water at 10 ml/min. The fractions containing the protein were pooled and lyophilized to yield protein as the acetate salt at 80 to 90% recovery. Residual TFA was shown to be <1% by ^19^F nuclear magnetic resonance (NMR).

### Synthesis of the DyLight 650-tagged Omomyc covalent dimer.

The Omomyc covalent dimer was reconstituted in PBS to 2 mg/ml, and the DyLight 650 label (Thermo-Fisher, Waltham, MA) was reconstituted to 10 μg/μl in DMF. A total of 15 μg label/mg Omomyc was added, and the mixture was allowed to react for 1 h at room temperature. Free dye was removed, and the Omomyc-DyLight 650 (Omomyc-650) stock was concentrated by repeated cycles of filtration in a 10-kDa Amicon Ultra-15 unit (Millipore Burlington, MA), made sterile with a Vivaspin 0.2-μm filter tube, and stored at 4°C until use. The concentration of the labeled stock was 5.22 mg/ml, and the degree of labeling was 0.3

### DNA binding assays.

For the fluorescence polarization assay, 100 μM 5′-FITC-labeled E box (5′-CCCCCACCACGTGGTGCCTGA-3′) DNA was diluted to 2 nM in gel shift buffer (10 mM Tris-HCl, 50 mM KCl, 1 mM EDTA, 5 mM MgCl_2_, and 10 mM DTT), and 20 μl was added to each well of a 384-well plate. Next, 20 μl Omomyc, MYC, or other proteins was added and incubated with the DNA for 30 min before reading of plates on a Wallac 1420 multilabel counter (Perkin-Elmer, Norwalk, CT) using the fluorescence polarization protocol. A similar assay was performed with the following 5′-6-carboxyfluorescein (FAM)-labeled noncanonical E box DNA sequences (boldface indicates changes from the wild-type E-box): VEGFA-1 5′-CCCCCACCAC**C**TGGTGCCTGA-3′ for VEGFA-1 and 5′-CCCCCACCACG**C**GGTGCCTGA-3′ for VEGFA-2.

### CHIP and ReCHIP assays.

Both CHIP and ReCHIP assays were performed using either a high-sensitivity CHIP kit (Active Motif, Carlsbad, CA) or ReCHIP kit (Active Motif). HCT116 cells were treated with 2.5 μM Omomyc in the presence of ProteoJuice; at 24 h, cells were fixed with 3% formaldehyde (Sigma, St. Louis, MO) and washed twice in cold PBS; and the cell pellet was then lysed using chromatin precipitation buffer from the kit and a Dounce homogenizer. After lysis, the pellet was collected from the lysis buffer and resuspended in CHIP buffer from the kit, and the chromatin was then sheared by sonication (Active Motif, Carlsbad, CA). A total of 25 μl of sheared chromatin was taken as an input sample, and the remaining chromatin was subjected to chromatin immunoprecipitation. Antibodies used for immunoprecipitation are indicated in Table SI in the supplemental material, along with protein G magnetic beads and streptavidin magnetic beads (Thermo-Fisher, Pittsburgh, PA). Samples were incubated overnight and then washed using wash buffer from the kit with a DynaMag magnet (Thermo-Fisher, Waltham, MA). Once eluted from beads, the input and immunoprecipitation samples were digested with proteinase K according to the kit directions, and the DNA was then purified using a QIAquick PCR purification kit (Qiagen, Carlsbad, CA). After being isolated and purified, chromatin samples were subjected to quantitative PCR (Q-PCR) using Sybr green (Thermo-Fisher). Genes used are indicated in Table SI. The percent input was calculated, with the means and SD calculated and graphed in Excel. Statistical significance was calculated using two-tailed unpaired Student’s *t* test, using Prism software (GraphPad).

For ReCHIP assays, HCT116 cells were treated with 2.5 μM Omomyc in the presence of ProteoJuice and treated as described above for the CHIP. After the first elution, the DNA was immunoprecipitated a second time according to the instructions of the ReCHIP kit, with the DNA being isolated a second time, treated with proteinase K, purified, and then subjected to Q-PCR with Sybr green using the primers indicated above. The percent input was calculated and graphed as described above.

### E box DNA-coupled bead mass spectrometry assay.

All pulldown experiments were performed in 96-well filter plates (Microlute filter plate, catalog no. 240002; Dunn), and liquids were passed through by centrifugation at 1,200 rpm. The cell lysates were diluted with equal volumes of compound pulldown (CP) buffer (50 mM Tris-HCl [pH 7.5], 5% glycerol, 1.5 mM MgCl_2_, 150 mM NaCl, 1 mM DTT), 1× protease inhibitor (SigmaFast protease inhibitor tablet; Sigma-Aldrich), 1× phosphatase inhibitors (phosphatase inhibitor cocktail; Sigma-Aldrich), and 1 mM DTT. Two milligrams of the lysate (CP buffer containing 0.8% IGEPAL) was used per pulldown experiment. The free Omomyc monomer was spiked into 0.5 ml of the lysate at increasing concentrations (DMSO vehicle; 3 nM, 10 nM, 30 nM, 100 nM, 300 nM, 1 μM, 3 μM, and 30 μM) and incubated for 60 min at 4°C with rotation in an end-over-end shaker. In parallel, 0.3 nmol of a 5′-biotinylated MYC E box (BtnTg) double-stranded DNA (dsDNA) sequence (BtnTg-5′-CCCCCACCACGTGGTGCCTGA-3′ annealed with 5′-TCAGGCACCACGTGGTGGGGG-3′; custom synthesized by Sigma-Aldrich) dissolved in PBS (pH 7.2) was incubated with 30 μl (1:1 slurry) of streptavidin beads (Pierce) for 1 h at room temperature. Subsequently, the beads were added to the Omomyc-treated lysate and incubated for 60 min at 4°C with rotation in an end-over-end shaker. The beads were then washed (1 ml of CP buffer containing 0.4% IGEPAL followed by 1 ml of CP buffer containing 0.2% IGEPAL and 1 ml of CP buffer containing 0.4% IGEPAL) and incubated in a solution containing 60 μl of 8 M urea and 10 mM DTT in 50 mM Tris-HCl (pH 8.0) for 20 min at RT to denature and reduce bound proteins. Proteins were then alkylated by the addition of 25 mM 3′-indoleacetic acid and incubation for 20 min in the dark. The urea concentration was reduced to 4 M by the addition of an equal volume of Tris-HCl (pH 8.0), and proteins were digested by the addition of LysC-trypsin (Promega) at an enzyme-to-protein ratio of ∼1:20 and incubation for 1 h at 37°C with shaking at 700 rpm. After transferring the digest into a 96-deep-well plate and reducing the urea concentration down to 1.2 M, digestion was continued overnight. The next day, digestion was stopped by acidification, and peptides were cleaned and desalted using a liquid handler (AssayMAP Bravo system; Agilent Technologies) with C_18_ cartridges (catalog no. 5190-6532; Agilent Technologies) according to a standard protocol provided by the manufacturer. Finally, peptides were eluted in a 96-well plate, dried down, and stored at −80°C.

Dissolved peptides (0.5% formic acid [FA]) were delivered to an Acclaim PepMap 100 C_18_ trap column (100-μm internal diameter [ID] by 2-cm length, 5-μm particle size; Thermo Scientific) at a flow rate of 5 μl/min in 100% solvent A (0.1% FA in HPLC-grade water). After 10 min of loading and washing, peptides were transferred to an analytical column (Easy-Spray, 75-μm ID by 50 cm, 2-μm particle size; Thermo Scientific) and separated at a flow rate of 300 nl/min using a 70-min nonlinear gradient ranging from 3% to 40% solvent B (0.1% FA in HPLC-grade ACN). The mass spectrometer was operated in data-dependent mode, automatically switching between MS and MS2 spectra. MS1 spectra were acquired over a mass-to-charge ratio (*m/z*) range of *m/z* 350 to 1,600 at a resolution of 60,000 in an Orbitrap instrument using an automatic gain control (AGC) target value of 3e6 with a maximum injection time of 50 ms. Up to 20 peptide precursors were selected for fragmentation by higher-energy collision-induced dissociation (HCD), with an isolation width of 1.2 Th, a maximum injection time of 100 ms, an AGC value of 1e5, 27% normalized collision energy (NCE), and 7,500 resolutions. Dynamic exclusion was set to 20 s, and singly charged precursors were excluded.

Peptide and protein identification and quantification were performed using MaxQuant version 1.6.0.13 (45). MS2 data were searched against the Swiss-Prot reference database (human proteins, with 42,233 entries, including the Omomyc sequence, which was added manually; downloaded 16 August 2017) using the embedded search engine Andromeda (46). Carbamidomethylated cysteine was set as a fixed modification; oxidation of methionine and N-terminal protein acetylation were set as variable modifications. Trypsin/P was specified as the proteolytic enzyme, and up to two missed cleavage sites were allowed. The fragment ion tolerance was set to 20 ppm, and the matching-between-runs option (0.7-min match time window) was enabled. Search results were filtered for a minimum peptide length of 7 amino acids, with a 1% peptide and protein false discovery rate (FDR). Common contaminants and reverse identifications were filtered out. Statistical data analysis and filtering were done using Perseus software (47). Curve fitting was carried out in GraphPad Prism 7.02 using a four-parameter nonlinear regression fit.

### Cellular thermal shift assay.

Ramos cells were harvested, washed with PBS, and then resuspended in cellular thermal shift assay (CETSA) lysis buffer (PBS supplemented with 0.4% NP-40, 5 mM DTT, and 1× Halt protease and phosphatase inhibitor cocktail [Thermo-Fisher Scientific, Waltham, MA]). Cell suspensions were lysed by sonication (Q700; Qsonica, Newtown, CT) for 4 cycles of 30 s each at an amplitude of 75, with 2 s on and 2 s off. The soluble fraction (lysate) was separated from cell debris by centrifugation at 18,000 × *g* for 10 min at 4°C. The cell lysate was diluted with CETSA lysis buffer to an approximate total protein concentration of 3 mg/ml and then divided into aliquots. For the *T*_agg_ determination experiment, aliquots of 0.9 ml each were incubated with either PBS or Omomyc (20 μM) for 2 h at 4°C. Each sample was then divided into 10 80-μl aliquots and placed into PCR tubes that were heated in a thermal cycler for 3 min at the specified temperatures in the range of 39°C to 67°C. For the ITDRF experiment, the cell lysate was first aliquoted into PCR tubes (80-μl total volume), incubated with different concentrations of Omomyc for 2 h at 4°C, and then heated at 54°C for 3 min. For all samples, upon cooling for 3 min to room temperature, insoluble proteins were separated by centrifugation at 20,000 × *g* for 20 min at 4°C. A volume of 20 μl of each supernatant was mixed with 1× NuPAGE LDS sample buffer (Thermo-Fisher Scientific, Waltham, MA), heated at 90°C for 10 min, and then analyzed by SDS-PAGE followed by Western blot analysis for the detection of Myc. Protein bands in the membranes were quantified by using ImageLab 5.2.1. Data were analyzed in GraphPad Prism 7, using the Boltzmann sigmoidal equation for *T*_agg_ calculations and the four-parameter logistic curve for ITDRF EC_50_ values.

### Proximity ligation assay.

A proximity ligation assay (PLA) was performed using a Duo-Link orange kit (Sigma, St. Louis, MO). HCT116 cells were treated with or without 10 μM Omomyc for 24 h, fixed with 4% formaldehyde (Sigma, St. Louis, MO), and then permeabilized using PBS (Thermo-Fisher) containing 1% bovine serum albumin (BSA) and 0.1% Triton X-100. The cells were blocked and treated according to the instructions of the Duo-Link kit (Sigma, St. Louis, MO). Samples were visualized using a Perkin-Elmer (Waltham, MA) Opera Phenix high-content imager at a ×40 magnification.

### Superresolution microscopy.

HCT116 cells were plated onto poly-d-lysine-coated glass-bottom plates (Greiner BioOne, Monroe, NC) at 20,000 cells per well. The cells were then treated with or without 10 μM Omomyc for 24 h, fixed with cold methanol (Thermo-Fisher), and then permeabilized using PBS (Thermo-Fisher) containing 1% BSA and 0.1% Triton X-100. Next, the cells were blocked in PBS-BSA-Triton X-100 buffer with 0.1% horse serum (Sigma) added. Blocked cells were treated with primary antibodies to UBF (Sigma), histone H3 (Active Motif), and Max (Sigma) at 1:500 dilutions for all antibodies. Cells were washed and then stained with goat anti-rabbit IgG Atto488 (Sigma) or goat anti-mouse IgG Alexa 568 (Thermo-Fisher) secondary antibody at a 1:1,000 dilution as well as the nuclear dye Hoechst 33342 at a 1:5,000 dilution. Cells were visualized using a Nikon SIM-E superresolution microscope with a 100× objective.

### RNA immunoprecipitation assay.

HCT116 cells were plated at 20 million cells per 15-cm^2^ plate overnight and then treated with ProteoJuice and 10 μM biotinylated Omomyc as described above. An RNA immunoprecipitation (RIP) assay was performed with a Magna-RIP RNA binding protein kit (Millipore-Sigma, Burlington, MA) according to the kit directions. Cells were scraped off the monolayer in ice-cold PBS and then lysed in RIP lysis buffer from the kit. The lysate was added to protein G and streptavidin beads along with the antibodies indicated (Table SI) overnight. Antibody-bead complexes were then washed, resuspended in proteinase K buffer, and subjected to heat treatment at 55°C for 30 min. After heat treatment, the bead mixture was placed on a magnet, the subsequent supernatant was removed, and the RNA was isolated using an RNeasy kit from Qiagen. After isolation, cDNA was generated from the isolated RNA as described above. RT-PCR was then performed as described above. Primer/probe sets are indicated in Table SI. The percent input was calculated as described above.

### Translating ribosome affinity purification assay.

The translating ribosome affinity purification (TRAP) assay was performed according to methods described previously by Heiman et al. (48), with minor modifications. HCT116 cells were transiently transfected with pCMV6-AC-GFP or pCMV6-AC-GFP-RPL10 (Origene, Rockville, MD) using Lipofectamine 2000 (Thermo-Fisher, Waltham, MA). Cells were grown overnight and then transfected with biotinylated Omomyc using ProteoJuice as described above. Fifteen minutes prior to lysis, 200 μg/ml of cycloheximide (Cayman Chemicals, Ann Arbor, MI) was added to cells. Cells were then washed twice in ice-cold PBS and then incubated and lysed in low-salt buffer (LSB) (20 mM HEPES-KOH [pH 7.5], 150 mM KCl, 10 mM MgCl_2_, and 1% NP-40 plus Halt EDTA-free protease inhibitors [Thermo-Fisher, Waltham, MA], 0.5 mM DTT, 100 μg/ml cycloheximide, and 10 μl/ml rRNasin [Promega, Madison, WI]). Cells were then homogenized and centrifuged at 2,000 × *g* for 10 min. The supernatant was then taken, and a 1/9 volume of 1,2-diheptanoyl-*sn*-glycero-3-phosphocholine (DHPC) was added to the supernatants. The mixture was then centrifuged at 20,000 × *g* for 10 min, and the subsequent supernatants were then used for affinity purification with anti-GFP antibody (Table SII), using protein G Dynabeads (Thermo-Fisher) overnight. Beads were then washed five times in LSB and then treated with 1× Tris-glycine SDS-PAGE loading buffer. Samples were subsequently subjected to SDS-PAGE and Western blotting as described above.

### Tissue distribution study.

Female BALB/c mice were given a single i.v. dose at 5.22 mg/kg. Terminal plasma samples were collected at 5 min, 15 min, 30 min, 1 h, 2 h, 6 h, 1 day, 2 days, and 3 days. Tissue lysates and blood were collected at 5 min, 2 h, 1 day, and 3 days. Standard titrations of Omomyc-650 were made in untreated, diluted blood, plasma, and liver lysates. Sample dilutions, dosing retainer dilutions, and standard titrations were added to black polystyrene 96-well plates (Corning, Corning, NY), and total fluorescence was read on a Promega GloMax microplate multimode reader. Concentrations for samples and dosing retainers were interpolated to the corresponding standard titration via a linear regression in GraphPad Prism.

### CAFÉ analysis.

Capillary fluorescent electrophoresis (CAFÉ) analysis was performed according to methods described previously by Piparia et al. and Brunn et al. (49, 50). Tissue lysates and plasma samples were diluted in sample buffer (HT Pico protein express kit; Perkin-Elmer, Waltham, MA) with 10 nM *N*-ethylmaleimide (Chem-Impex, Inc., Wood Dale, IL) and added to 96-well skirted PCR plates (Thermo-Fisher, Waltham, MA). Dilutions were heated by using a thermal cycler (C1000 Touch; Bio-Rad, Hercules, CA) for a single 10-min cycle at 80°C and run on a LabChip GXII instrument with an HT Pico protein express chip (Perkin-Elmer, Waltham, MA). Peak area analysis was performed using WinNonlin (Pharsight, Mountain View, CA), and the total fluorescent peak area under the curve (AUC) within pertinent molecular weight ranges (2 to 16, 16 to 24, and 24 to 120 kDa) was determined and graphed using Prism software (GraphPad).

### Data availability.

RNA-Seq data were uploaded to the GEO repository under accession no. GSE131075.

## Supplementary Material

Supplemental file 1

Supplemental file 2
