# Supplementary material for: Omomyc Reveals New Mechanisms To Inhibit the MYC Oncogene
Source: Mol Cell Biol. 2019 Oct 28;39(22):e00248-19. doi: 10.1128/MCB.00248-19 (PMC6817756; doi:10.1128/MCB.00248-19)
Supplement: Supplemental file 1 [file MCB.00248-19-s0001.pdf]

Supporting Information

Supplemental Methods Table I: Antibodies Primer sets used in Q-PCR for CHIP and ReCHIP assays.used in experiments.

	**Antibodies used in this study**	
**Target**	**manufacturer/catalog #**	**assay**
**Myc**	**Abcam/ab3072**	**Western, PLA, Immunfluorescence, immunoprecipitation**
**Myc**	**Cell Signaling Technologies/9402**	**CHIP**
**Max**	**Sigma/WH0004149M1**	**Western, PLA, Immunfluorecence, CHIP, immunoprecipitation**
**Biotin**	**Abcam/ab1227**	**Western, PLA, Immunfluorecence, CHIP, immunoprecipitation**
**penta-His tag**	**Qiagen/35560**	**PLA**
**UBTF**	**Sigma/HPA006385**	**Immunofluorescence**
**Histone H3**	**Active Motif/39763**	**Immunofluorescence**
**WDR5**	**Cell Signaling**	**CHIP**

	Technologies/13105	
control IgG	Abcam/171870	CHIP
goat anti-Rabbit HRP	Cell Signaling Technologies/7074	Western
goat anti-Mouse HRP	Cell Signaling Technologies/7076	Western
	Primer/Probes used	
probe	Sequence	
Control F	tttctcacattgcccctgt	
Control R:	tcaatgctgtaccaggcaaa	
NPM 1 F	cacgcgaggtaagtctacg	
NPM 1R	ttcaccgggaagcatgg	
ARC F:	gctgggccaatgagaac	
ARC R:	agctctgcgctgagtcctg	
NCL F:	ctaccaccctcatctgaatcc	
NCL R:	ttgtctcgctgggaaagg	
FBXW8 F:	gtgataggcagcagagctga	
FBXW8 R:	tgtacgcacgtggtggtc	
BOP1 F:	tcctttaccccgcagtcc	
BOP1 R:	cagtgaatcggtcttgggc	
VEGFA F	agggggcttgctgtcact	
VEGFA R	cagcaatccaccccaaaa	
HSPBAP1 F	GCTCTCACGTGGAGGTCAC	
HSPBAP1 R	CACACCCGGGGTATCAAA	
FBX32 F	TGCTGTGGTTTAGCTTTGCC	
FBX23 R	TTGGCCTTCCGAATCTT	

	GCT	
PUS 1 F	CCTCGACTCCTGAGGAAAGC	
PUS1 R	ATCAACTCCAACGTCCCCTG	
PUS control F	GTCCGGCAGGATTTAGGTG	
PUS control R	CTGATGGAAAGCAGCAGGA	
RRS1 F	AGGTCCCGCTTCTAATCCCA	
RRS1 R	TTGCAACTTCTCTGCCTCGT	
LYAR F	GAACTCCTTCCTCCATGCGT	
LYAR R	CAGAAGCGAATTCTGCACCG	
LYAR Control F	GAATCGAACTTATTTGCATACGG	
LYAR control R	TCCCCTTAAAAGGACAATAGAGG	
rDNA, transcribed region 45S F H1 amplicon	GGCGGTTTGAGTGAGACGAGA	
rDNA, transcribed region 45S R H1 amplicon	ACGTGCGCTCACCGAGAGCAG	
rDNA, transcribed region, 18S H4 amplicon	CGACGACCCATTCGAACGTCT	
rDNA, transcribed region, 18S H4 amplicon	CTCTCCGGAATCGAACCCTGA	
rDNA transcribed region, 5.8S H8	AGTCGGGTTGCTTGGG	

**amplicon**	**AATGC**	
**rDNA transcribed region, 5.8S H8 amplicon**	**CCCTTACGGTACTTGTT GACT**	
**rDNA, non-transcribed region H13 amplicon**	**ACCTGGCGCTAAACCAT TCGT**	
**rDNA, non-transcribed region H13 amplicon**	**GGACAAACCCTTGTGTC GAGG**	

Supplemental Table II: Excel file of fold expression of genes affected by Omomyc treatment in HCT116 cell.
